# Implementing the flipped classroom model to enhance knowledge retention in pharmacology: a local case study at Semmelweis university

**DOI:** 10.1186/s12909-025-06913-5

**Published:** 2025-02-28

**Authors:** Zsófia Onódi, Pál Riba, Péter Ferdinandy, Anikó Görbe, Zoltán V. Varga

**Affiliations:** 1 Department of Pharmacology and Pharmacotherapy, Semmelweis University, Nagyvárad tér 4, Budapest, H-1089 Hungary; 2 Center of Pharmacology and Drug Research & Development, Semmelweis University, Budapest, Hungary

**Keywords:** Flipped classroom, Active learning, Blended learning, Pharmacology, Medical education

## Abstract

**Background:**

The flipped classroom (FC) approach has demonstrated efficacy in enhancing the learning process, including within higher education contexts such as medical education. Recently, FC has emerged as potential alternative to traditional teaching models across various disciplines, particularly due to its more engaging nature. However, there is limited data available regarding its impact on student performance, particularly in the context of long-term knowledge retention in pharmacology. In this study, our objective was to assess the short- and long-term impact of FC on student performance in Hungarian pharmacology teaching at medical faculty in Semmelweis University (Budapest, Hungary).

**Methods and results:**

161 medical students and 10 teachers were involved in this study. We flipped four seminars, then we assessed the academic performance by using multiple choice tests immediately and two weeks after the flipped class. A follow-up assessment was conducted six months after the initial two FC sessions. Our findings indicated that the FC approach enhanced both short- and long-term knowledge retention across most topics. Notably, this long-term improvement was evident even six months after the original seminars on specific subjects. However, despite these topic-specific benefits, the overall performance, including exam grades, did not show significant improvement when compared to the conventional teaching approach. Additionally, we assessed student and teacher perceptions using two questionnaires immediately after practice and at the end of the year. According to the questionnaire responses, students perceived positively the FC approach, emphasizing its interactive and thought-provoking aspects. However, they identified the time-consuming nature of preparation as a significant concern. Teachers also viewed the FC approach favorably, particularly appreciating its interactivity and potential for greater effectiveness. Interestingly, more experienced teachers were less receptive to the FC method, and their perceptions of it were less favorable compared to those of their younger colleagues.

**Conclusions:**

The flipped classroom approach presents a viable strategy for teaching pharmacology, with the potential to enhance student performance and engagement. However, student occupation and faculty resistance may pose significant challenges to the implementation of such alternative teaching methods.

**Supplementary Information:**

The online version contains supplementary material available at 10.1186/s12909-025-06913-5.

## Introduction

Pharmacology, a traditional subject dealing with general pharmacological fields (e.g. pharmacodynamics, -kinetics or -genetics) and concepts (so-called core concepts [1]) as well as detailed characterization of clinically used medicines and drugs. There are various approaches to teaching pharmacology for medical students, which vary across universities based on their curricula. Some institutions integrate pharmacology into clinical subjects, utilizing case-based learning [2, 3], while others, such as our university, offer it as an individual subject. Although numerous drug databases are accessible for medical professionals as reference on different pharmacological agents, possessing detailed and long-term knowledge of the most critical features of commonly used drugs remains essential. Thus, effective methods that facilitate long-term knowledge retention of pharmacology are needed.

Alternative teaching approaches including interactive and skill-based methods are under consideration for being introduced into medical education as they are supposed to enhance active learning [4]. Flipped classroom is a well-established teaching method which is based on the flip of the traditional approach: students should prepare before the class using online material (instead of listening to the material passively during seminars), and the active learning process (which is usually after the seminar) is placed in the classroom, when they actively work with tasks [5].

During COVID19, both teachers and students gained a significant amount of experience with online learning. Although face-to-face learning might be more effective than online teaching [6, 7], the combination of online and offline learning (i.e. blended learning) gives new opportunities to enhance effectiveness of teaching. Some authors report favorable results on the efficacy of flipped classroom in medical education particularly on student perception [4, 8]. Furthermore, it was reported that flipped classroom approach might improve student performance [9, 10]. However, it was reported that flipped classroom may decrease long-term knowledge retention in pharmacology-related subjects [11, 12]. Still, further comprehensive data is needed on certain aspects of the efficacy of flipped classroom in pharmacology teaching especially its effect on short- and long-term knowledge retention for medical students.

Therefore, the major objectives of this present study were: (1) to test whether flipped classroom can improve short-term student performance compared to conventional frontal teaching method, (2) to examine the long-term knowledge retention of pharmacology by flipped classroom method, and (3) to assess the student and teacher perception on the new method at Semmelweis University.

## Methods

### Framework of the study

#### Student and teacher participants

A total of 161 Hungarian students and 10 teachers participated in the study. Participation was voluntary; however, students did not choose their instructional method. Instead, group assignments were determined by their teachers’ preferences. Teachers who favored the traditional lecture-based method had all their students assigned to the control (CON) group, while those preferring the flipped classroom (FC) approach had all their students assigned to the FC group.

The gender distribution was slightly skewed, with more females in both groups (51 in CON, 41 in FC; Suppl. Tabl. 1). Additional demographic data, such as ethnicity and age, were not collected, as the Hungarian population is largely homogeneous, with most individuals identifying as white or having a slightly mixed background. Along the same line, most of the students are within their early twenties.

The study included 10 teachers, with 6 assigned to the control (CON) group and 4 to the flipped classroom (FC) group (Suppl. Table 2.) The median age of teachers was similar in both groups (31.5 years in CON and 31 years in FC). In terms of gender distribution, the CON group had a higher proportion of male teachers (5 males, 1 female), while the FC group had an equal number of male and female teachers (2 males, 2 females). Both groups had comparable teaching experience, with 2 teachers in each group having less than two years of experience and 2 teachers in each group having more than five years of experience.

To ensure participant anonymity, students included in the study were assigned unique identification codes that were independent of their name, age, or gender. However, their test results were not anonymized, as individual performance needed to be tracked for analysis. Importantly, access to the data was strictly limited; no other teachers, external colleagues, or third parties had access. Only the authors had unrestricted access to the data, ensuring confidentiality and data protection throughout the study.

#### Annotation of pharmacology course

The target course was Pharmacology, a two-semester course for third-year medical students. Our approach in the Pharmacology course focuses on the theoretical background of pharmacology, covering drug mechanisms of action, indications, side effects, and other key characteristics essential for future clinical practice. To build on this knowledge, students receive further training in the following year at the Clinical Pharmacology course, which discusses the use of medications in clinical settings. To help students bridge the gap between theory and practice, the Clinical Pharmacology course has been established in an interactive approach (i.e. team- and case-based learning), focusing on the application of pharmacological agents and guideline-based therapies in real-life clinical situations.

#### Structure of pharmacology course

It includes weekly lectures (90 min) and group seminars (105 min with a 10-min break) during the semesters, covering all major pharmacology topics with minimal overlap between the two formats. Lectures are traditional presentations for 300–400 students at personal class with limited possibility to interact with students. Traditional seminars share a similar structure as lectures in a smaller group format (maximum of 25 students). These consist of 90-minute long frontal presentations with opportunities for students to ask questions and discuss topics from lectures or previous seminars. However, the number of questions that can be addressed is limited due to time constraints. Each group is usually guided by one or two teachers throughout the two semesters. To provide students with feedback on their progress during the semesters, mid-semester tests (“midterm tests”), based on multiple choice questions, are performed to cover topics studied up to that point. Midterm tests have no minimal threshold; however, students can receive benefits for strong performance, such as having fewer questions to answer during exams. Both semesters finalized with classic oral exams at which students need to identify five substances, then answer a series of questions on (1) a pharmacology core concept with practical examples, and (2) three different groups of compounds (e.g. penicillins, calcium channel blockers, antimetabolite cytotoxic drugs).

#### Study design and timeline

**Fig. 1:**
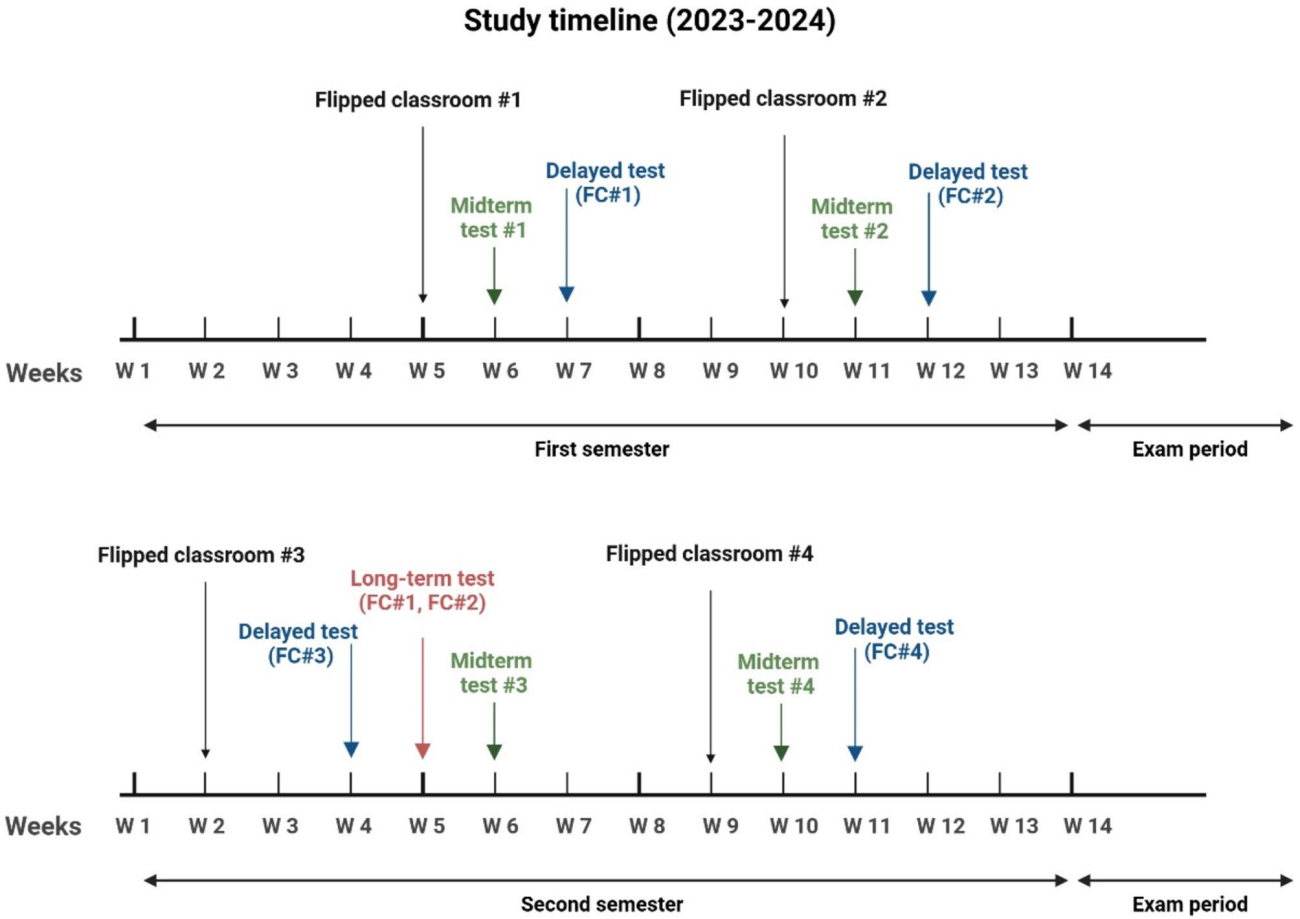
Study design and timeline of investigating the effects of flipped classroom approach in pharmacology teaching

As part of this investigation, we flipped two seminars each semester, up to a total of four seminars (Fig. 1). Conventional and FC seminars were conducted within the same week to ensure time-matched evaluation and to prevent the unintentional release and discussion of questions. The flipped seminars were held at week 5 and 10 in the first, and week 2 and 9 in the second semester. Detailed presentations on theoretical background of each topic were available for all the teachers involved in the study to provide the materials for preparation irrespective of having conventional or flipped classroom. Students were informed on this study and flipped classroom seminars to provide them with time for pre-seminar preparation. All students have unlimited access to the presentation slides related to the flipped topics. Immediately and two weeks after the seminars, students completed multiple question tests and questionnaires to gain information on their short- or long-term performance and feedback. Midterm tests were performed in the week 6 and week 10–11. In the second semester, an extra test was inserted to measure the long-term performance later.

#### Design and strategy of our flipped classroom seminars

To introduce flipped classroom (FC), we identified the four different topics to flip. For each semester we chose (1) a short, easy-to-understand topic, and (2) a long, more difficult topic. “Pharmacology of gout and migraine” and “Positive inotropes” were chosen as topics for FC #1 and #3, respectively, as these topics contain relatively low number of compounds, and less complicated mechanisms. “Antiepileptics” and “Cytotoxic drugs” were chosen for FC #2 and #4, respectively, as both are considered difficult, complex topics with high number of drugs and many details to learn. We also revised the learning outcomes and highlighted the take-home-messages of each topic to develop our online and in-classroom materials based on them.

For flipping seminars, we removed frontal presentation parts from our in-person seminars, and prepared digital materials uploaded to online learning platform for preparation. The online material was accessible at least one week before the in-person seminar to leave the students enough time for preparation prior to in-person seminar. Briefly, online preparation materials contained (1) detailed slide show used by both conventional and FC teachers, (2) video material discussing the most important aspects of the topics with short multiple-choice questions in lesson format to monitor student activity, (3) supplementary materials such as relevant case studies from the literature. The videos were divided into 5-8-minute sessions in the lessons finalized with short questions to increase learning flexibility and enhance understanding by incorporating brief feedback.

The structure of the in-person seminar for flipped groups (105 min) was divided into two parts. In the first part (30–35 min), students had the opportunity to familiarize themselves with the structure of the whole seminar and tasks as well as we ensured students had access to digital equipment e.g. smartphone, tablet or notebook. As we assumed there would be students who might be less prepared from online material, we continued with a short task aiming to recall related theoretical knowledge in a gamified form such as searching for the relevant drug packages or filling encyclopedia articles with missing key words. In the next part (60–65 min), problem-oriented case reports were processed in group work. All the case reports contained tasks and questions concentrating on the most important pharmacological concepts and phenomena. During group work, students had the opportunity to discuss specific questions with their fellows and teacher. After completing the tasks, all cases were discussed in detail. Seminars were finished with immediate (“early”) tests and student feedback questionnaire for both control and flipped groups.

### Student performance

To evaluate student performance, we developed multiple-choice tests with a maximum score of 15 points. These tests comprised questions designed to assess knowledge in key areas: (1) fundamental principles and core concepts of pharmacology, (2) drug-specific details including mechanisms of action, indications, and adverse events, and (3) problem- and case-oriented scenarios. All test questions were reviewed and approved by the teachers involved in this study, irrespective of their teaching method. The tests were performed immediately after the flipped seminar (referred to as the “short-term test”) and two weeks later following the seminar (referred to as the “delayed test”). An additional delayed test was performed six months after the relevant seminars (FC #1 and #2) to evaluate the long-term efficacy of FC (referred to as the “long-term test”). Additionally, the results of midterm tests (a maximum score of 30 points) and final exam grades (1–5 scale) were also analyzed.

### Student and teacher perception

To assess student and teacher perception, we utilized different questionnaires containing Likert scale-based statements as well as open-ended questions. All the questionnaires were anonymous.

For students, questionnaire #1, performed immediately after the FC seminars, was the official 10-question Likert scale feedback commonly used at Semmelweis University (Table 1). This questionnaire was filled by students from both conventional and flipped groups. Questionnaire #2, administered at the end of the second semester only for flipped classroom students, included 17 Likert-scale questions and open-ended questions (Appendix 2).

To examine teachers’ perception, we developed a questionnaire that included both Likert scale-based, and open-ended questions investigating the general attitude towards teaching and flipped classroom (Appendix 3). This questionnaire was also completed at the end of the second semester by all teachers involved in traditional and flipped teaching methods.

**Table 1:** The differences of student perception of each topic between flipped or conventional classroom groups

Question	FC #1		FC #2		FC #3		FC #4	
	CON	FC	CON	FC	CON	FC	CON	FC
1. How logical and easy-to-understand was the instructor’s explanation?	4.64 ± 0.089	4.59 ± 0.109	4.77 ± 0.089	4.86 ± 0.053	**4.75** **± 0.078**	**4.96** ± 0.028*	4.84 ± 0.071	4.75 ± 0.086
2. How thought-provoking or problem solving-oriented was the instructor?	**4.43** **± 0.088**	**4.67** ± 0.091*	**4.42** **± 0.154**	**4.79** ± 0.071*	**4.49** **± 0.120**	**4.82** ± 0.062*	4.61 ± 0.128	4.63 ± 0.128
3. How practice-oriented and interactive were the lessons?	**3.89** **± 0.154**	**4.85** ± 0.063*	**4.10** **± 0.193**	**4.86** ± 0.063*	**4.16** **± 0.149**	**4.84** ± 0.060*	4.32 ± 0.185	4.68 ± 0.121
4. How organized and well-structured were the lessons?	4.66 ± 0.078	4.63 ± 0.114	4.71 ± 0.095	4.72 ± 0.084	4.69 ± 0.082	4.84 ± 0.060	4.87 ± 0.056	4.73 ± 0.095
5. How helpful was the instructor?	**4.93** **± 0.043**	**4.70** ± 0.119*	5.00 ± 0.000	4.98 ± 0.023	4.88 ± 0.053	4.94 ± 0.034	4.95 ± 0.037	4.95 ± 0.035
6. To what extent did you manage to master the given topics of the practical lessons?	3.61 ± 0.146	3.63 ± 0.189	3.77 ± 0.226	3.91 ± 0.169	4.28 ± 0.137	4.40 ± 0.095	4.18 ± 0.192	3.78 ± 0.191
7. To what extent were the activities during the lessons in line with the knowledge acquired during the lectures?	4.15 ± 0.162	3.88 ± 0.183	3.81 ± 0.264	4.16 ± 0.166	4.52 ± 0.119	4.32 ± 0.138	4.46 ± 0.167	4.40 ± 0.151
8. Evaluate your own activity throughout the lessons.	3.63 ± 0.179	3.97 ± 0.177	3.81 ± 0.234	4.21 ± 0.147	**4.06** **± 0.170**	**4.58** ± 0.081*	4.27 ± 0.184	4.39 ± 0.114
9. How useful did you find the time spent at the practical lessons?	4.13 ± 0.140	4.28 ± 0.143	4.07 ± 0.202	4.28 ± 0.157	4.41 ± 0.132	4.62 ± 0.094	4.26 ± 0.167	4.33 ± 0.154
10. Overall, how do you evaluate the instructor’s activities?	4.68 ± 0.073	4.70 ± 0.134	4.74 ± 0.080	4.84 ± 0.066	**4.76** **± 0.084**	**4.96** ± 0.028*	4.90 ± 0.050	4.88 ± 0.064

We summarized the descriptive statistics (e.g. median, mode) on the Likert-scale responses, and we performed Spearman correlation to find associations between statements. Open-ended questions were analyzed by two persons who read all the answers and assigned a maximum of five keywords to each of them, then discussed the findings. Then the frequency of repeating keywords was visualized by using a word cloud generator.

### Statistical analysis

Data is expressed as *mean ± standard error of mean* if not indicated otherwise. Comparisons of two groups were performed using the nonparametric Mann-Whitney-U test. Data with more than two groups was evaluated by Kruskal-Wallis test followed by Dunn’s multiple comparisons test. To detect differences between subcolumns, we performed chi-square test in nested model. *P* < 0.05 were considered statistically significant. Statistical analysis was performed with GraphPad Prism 8 (GraphPad Software Inc).

## Results

### Flipped classroom improved student performance in short term

**Fig. 2:**
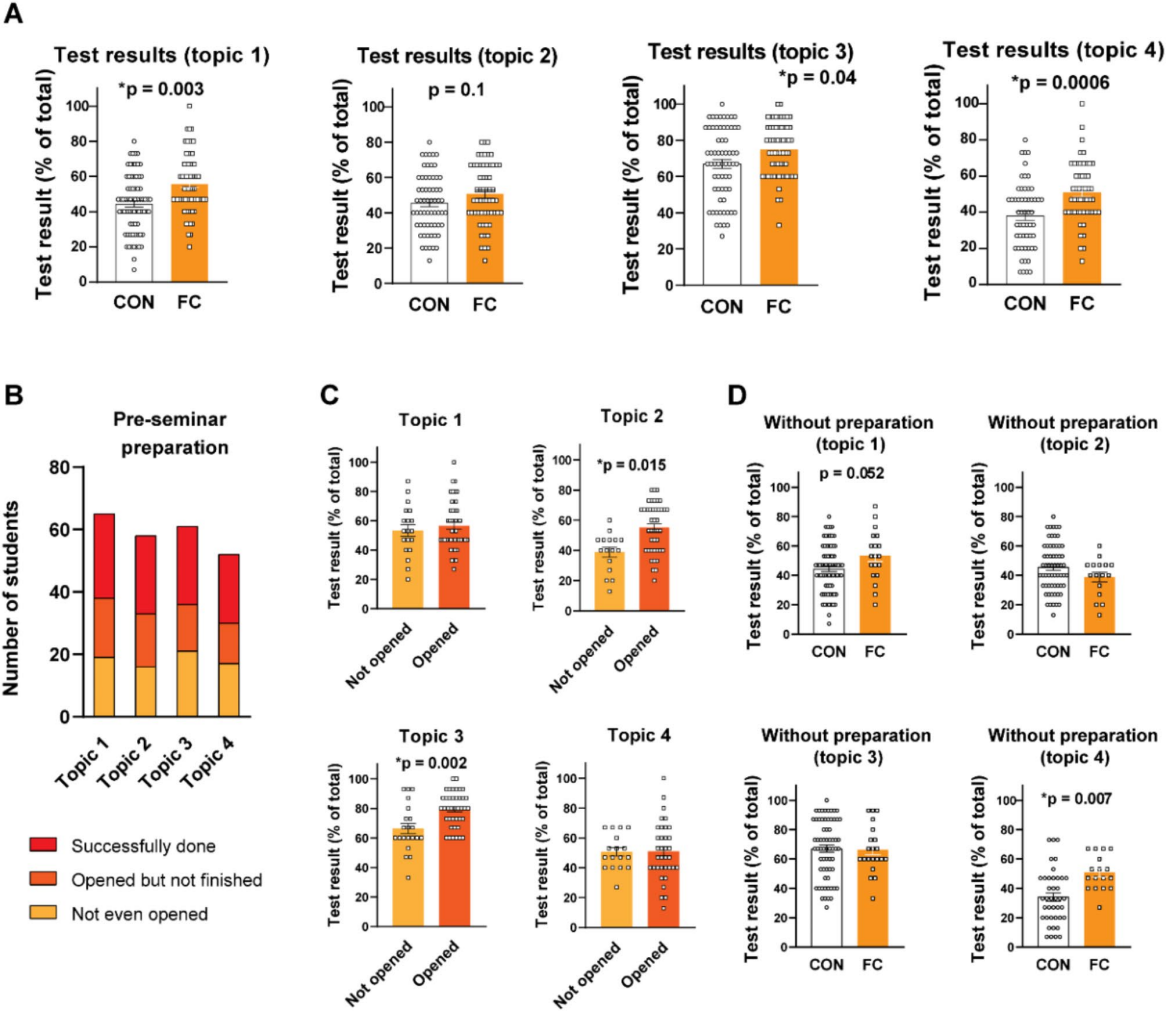
The impact of flipped classroom on topic-specific student performance in short-term. (**A**) The results of 15-question tests performed immediately after seminars. Each point represents one student. *P < 0.05 vs. CON, Mann-Whitney test; *n* = 52–72. (**B**) Pre-seminar preparation of the students expressing the number of students prepared or not prepared for the seminars. (**C**) The results of 15-question tests of students participating at flipped seminar based on their preparation. “Not opened” presents students who “not even opened” the Moodle material, while “Opened” represents students who successfully finished or at least opened the lesson. *P < 0.05 vs. CON, Mann-Whitney test; *n* = 16–42. (**D**) The results of 15-question tests of students participating at flipped seminar without significant preparation were compared to the conventional classroom results. *P < 0.05 vs. CON, Mann-Whitney test; *n* = 16–72. Table format of the original dataset can be found in Supplementary Tables 4–5

It is reported that flipped classroom approach has improved student performance in different fields including medical area [10]; however, less is known on the effects of flipped classroom in pharmacology teaching particularly involving multiple different topics. Therefore, we tested student performance immediately after each seminar with a 15-question test. As shown in Fig. 2A and Supplementary Table 4, a significant increase in the total points was observed in FC groups compared to CON groups in three topics (*p* = 0.003, *p* = 0.04, *p* = 0.0006; Fig. 2A; Suppl. Table 4). Interestingly, the results of FC #2 tests did not show significant improvement (*p* = 0.1; Fig. 2A “Test results (topic 2)”; Suppl. Table 4). When we analyzed the results by small study groups within the flipped or conventional seminars, we observed differences within the same teaching approach, suggesting the influence of factors independent of the teaching method (Suppl. Figure 1, Suppl. Table 4–7).

As a significant improvement by flipped classroom was demonstrated, we aimed to identify the role of pre-seminar preparation in this improvement. Flipped classroom demands preparation before the seminar, thus, we hypothesized that pre-seminar preparation may be the primary reason for the benefits of the approach rather than the on-site part of seminar. To assess this, we monitored student activity in the online learning platform prior to each seminar including the time they spent with the corresponding video as well as related their practice test results. We divided the students into three groups for a subgroup analysis after the seminars: (1) students, who did not open the lesson (assumed not to be or less prepared), (2) students, who opened the lesson but did not spend significant amount of time (20 min <) or/and did not finish the lesson, (3) students, who successfully finished the lesson (Fig. 1B-D, Suppl. Table 5). We found that the majority (FC #1: 70.77%, #2: 72.41%, #3: 65.58%; #4: 67.31%; Fig. 1B) of students spent time with preparation indicating their motivation to gain benefits from the practices (Fig. 2B). Surprisingly, we did not find a clear connection between pre-seminar preparation and test results in case of two topics as we observed no significant improvement among prepared students after FC #1 and FC #4 (Fig. 2C; Suppl Table 5). Along the same line, we investigated whether the lack of significant pre-seminar preparation negatively impacted student performance, a potential disadvantage previously identified with the flipped classroom approach [9, 13]. Interestingly, we found that flipped classrooms were noninferior compared to conventional classrooms, even for students who did not prepare prior to seminars (Fig. 2D).

### Flipped classroom improved student performance in the long term

**Fig. 3:**
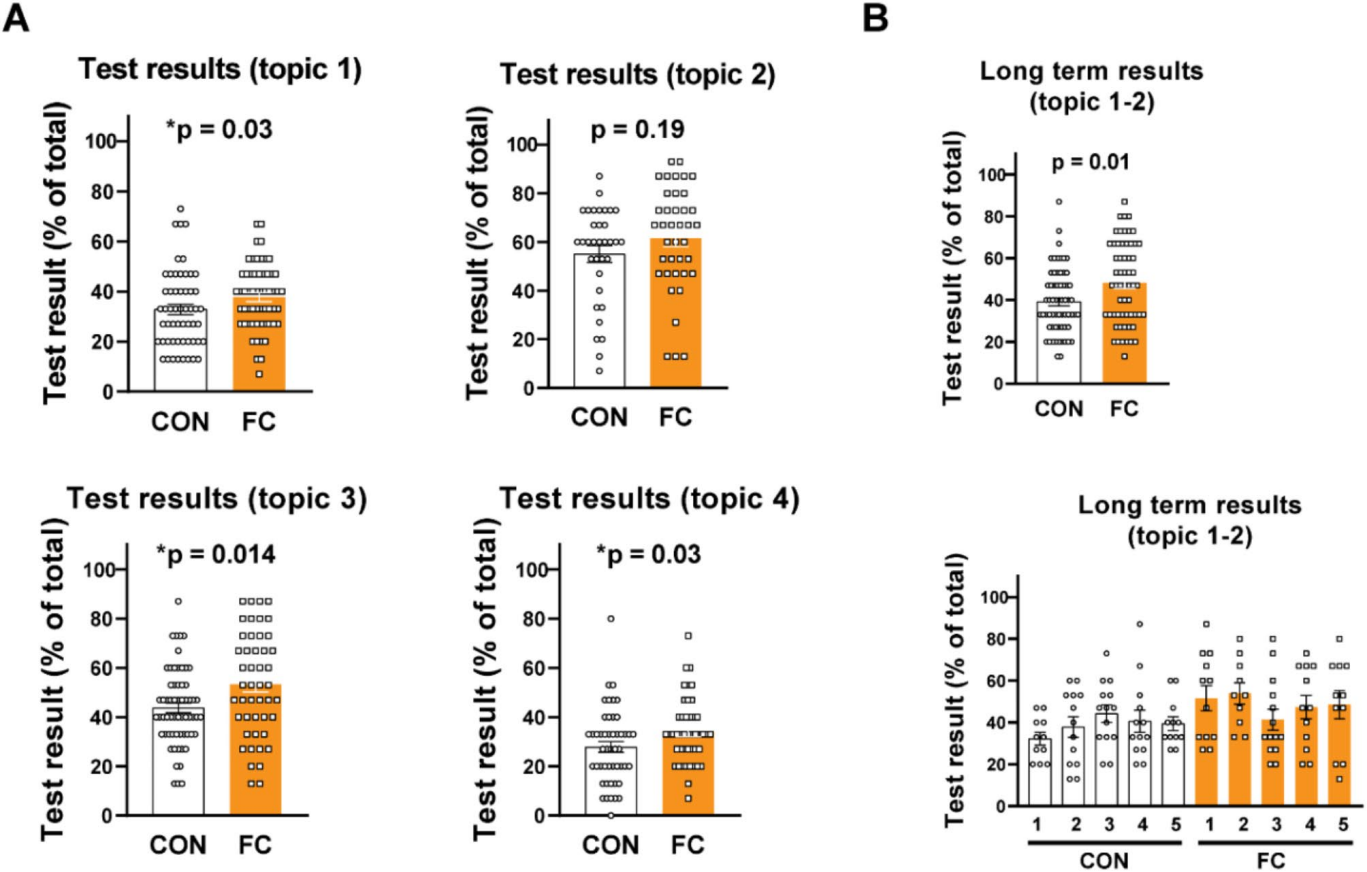
The impact of flipped classroom on topic-specific student performance. (**A**) The results of 15-question tests performed two weeks after seminars. Each point represents one student. *P < 0.05 vs. CON, Mann-Whitney test; *n* = 34–61. (**B**) The result of a 15-question test performed six months after the first two seminars (topic of FC #1 and 2) in a summarized (top) or a group-separated (bottom) form. Each point represents one student. *P < 0.05 vs. CON, Mann-Whitney test; *n* = 59–63. Table format of the original dataset can be found in Supplementary Tables 6–7

In medical education, long-term retention of knowledge is crucial for students. Although we observed significant improvement immediately after seminars, we have less data on the delayed and long-term performance of students receiving flipped seminars. Therefore, we aimed to investigate their delayed performance with another 15-question test performing two weeks after the corresponding seminars. Similarly to early tests, students showed overall better performance two weeks after the seminars except of FC #2 (FC #1 *p* = 0.03; #2 *p* = 0.19; #3 *p* = 0.014; #4 *p* = 0.03; Fig. 3A, Suppl. Table6), which was only a trend of improvement at both time point suggesting the presence of knowledge retention in most cases (Fig. 3A, Suppl. Figure 2; Suppl. Table6).

To further clarify the improvement, students performed another 15-question long-term test from topics of FC #1–2 in the second semester six months after the original flipped seminars. Surprisingly, students learning in the flipped setup reached higher points compared to conventional students long after the original seminars indicating the flipped classroom can facilitate long-term retention of knowledge in specific topics (median points of CON = 40 and FC = 47, *p* = 0.01; Fig. 3B, Suppl. Table 7).

### Flipped classroom did not alter student performance at midterm tests and exams

After confirming the short- and long-term effects of flipped classroom on specific topics which they learnt in the flipped structure, we further aimed to assess the overall student performance irrespective of the topics probing the hypothesis that flipped classroom may facilitate the knowledge on other fields indirectly. Thus, we analyzed the results of midterm tests performed during the semester, and the exams after each semester.

**Fig. 4:**
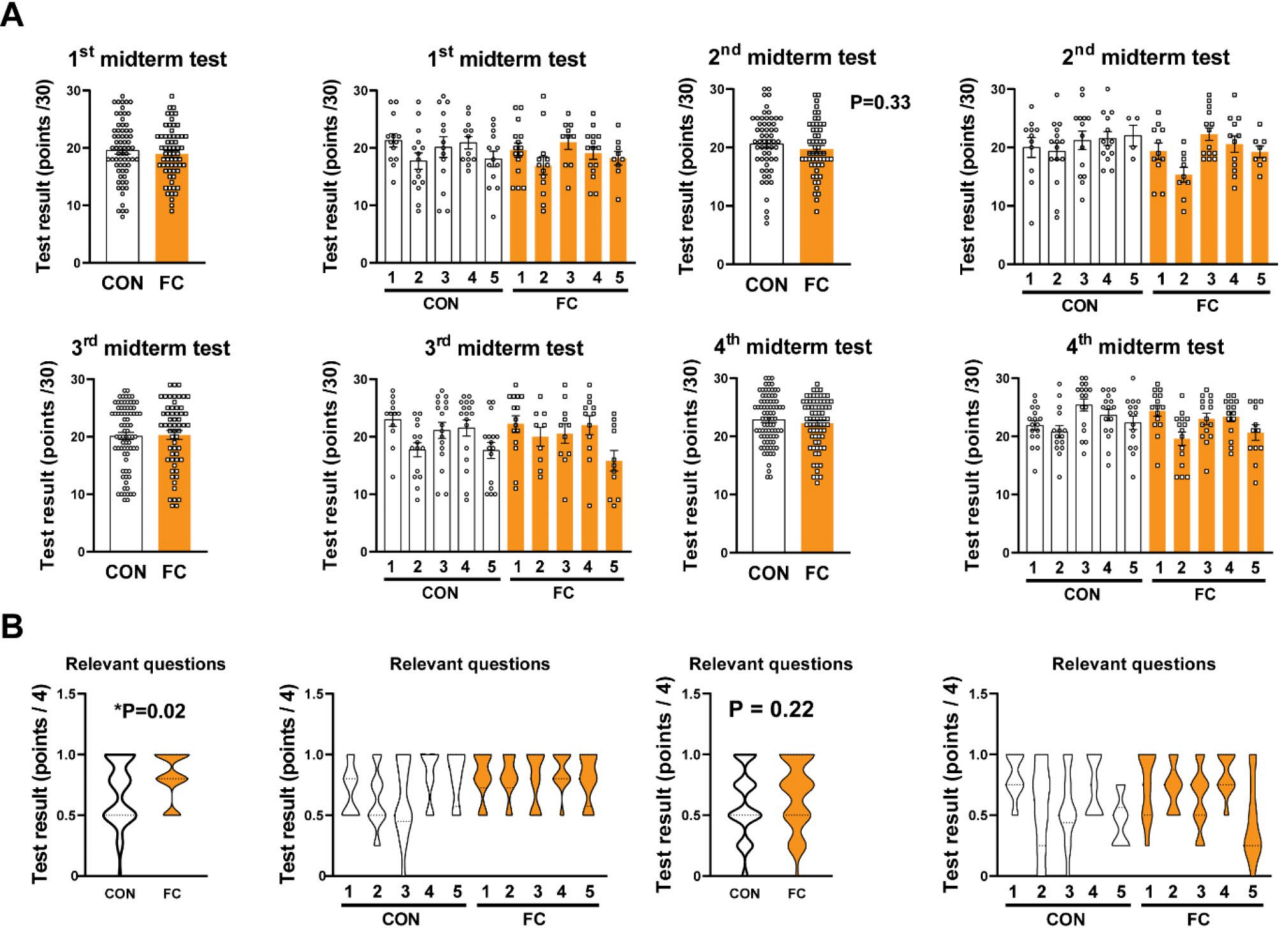
The impact of flipped classroom on overall student performance. (**A**) The results of midterm tests in a summarized and a group-separated form. Each point represents one student. *P* > 0.05 vs. CON, Mann-Whitney test; *n* = 62–80. (**B**) The results of relevant questions from 2nd and 3rd midterm tests in a summarized and a group-separated form. Each point represents one student. *P < 0.05 vs. CON, Mann-Whitney test; *n* = 48–62

No significant changes were found between the students learning pharmacology in different setups (Fig. 4A). As the first midterm test was performed a week after the first flipped seminar, and no flipped topic-related questions were included, the first midterm test might be representative of the baseline. The results of the first midterm test indicate that no significant differences can be found among the groups at the baseline time point; however, small, tendentious differences might be observed between some groups (Fig. 4A). As for the midterm tests after the FC #2 and FC #3, four relevant questions were included which were randomly picked from a larger questions pool for each student. Thus, we analyzed the results of the relevant midterm questions (Fig. 4B). Surprisingly, significant improvement was found in the case of the second midterm test which contained questions relevant from the topic of FC #2, however, only a trend of improvement was found in case of the third midterm test (questions from FC #3; Fig. 4B). Additionally, we observed highly variable performance among the groups, possibly as a consequence of low number of relevant questions. Similarly to midterm tests, students learning in flipped class show no improvement according to the grade they received at the end of the first or second semester (Suppl. Figure 3.)

### Students perceived flipped classroom positively

Flipped classroom has been reported to be approved by students widely because of its engaging form, however, issues may be raised regarding the preparation and student responsibility in it [14, 15]. Thus, we aimed to assess student perception on our pharmacology-oriented flipped classroom with two different questionnaires.

Statistical comparison of the grades given by the students immediately after seminars according to the questions above. Asterisk(*) indicates significant difference compared to students learning in conventional classroom. Data are expressed as mean **±** standard error of mean. *P < 0.05 vs. CON, Mann-Whitney test; *n* = 31–56.

The short questionnaire completed by the students immediately after the corresponding seminar contained direct questions about students’ opinion regarding the structure, the interactivity and usefulness of the seminar as well as the activity of the teacher (Table 1). According to the data collected, students learning in flipped class perceived better interactivity and problem-oriented approach during seminar compared to students learning in the conventional class. Surprisingly, flipped classroom students may perceive their teachers less helpful as we observed a significant decrease in the grades they gave (Table 1). The students perceived the FC #3 class as the best based on the feedback they gave. However, they felt less confident on FC #4, as a tendentious increase in Q3 and a decrease in Q6 were found (Tabl. 1).

Next, we asked feedback from the students learning in flipped classroom to assess the flipped course at the end of the second semester with a long Likert-scale based anonymous questionnaire with five open ended questions (Table 2). We analyzed the data by Spearman correlation to find potential association. We found a positive correlation between the number of classes students attended (FC No) and the feeling that the course helped with learning pharmacology (Q12; Table. 2). Unsurprisingly, students who felt flipped classroom more engaging (Q1; answer: 4–5 / agree-strongly agree; *n* = 25), helpful (Q12) and motivating (Q16) also would prefer to have the entire course in a flipped classroom setup. These students also preferred preparing at their own pace (Q9) with online material (Q11). In addition, these students might be more motivated to use the pre-seminar preparation material in time (Q8). We also identified a small number of students who felt less confident with flipped classroom and self-pacing learning (Q9; answer: 4–5 / agree-strongly agree; *n* = 3); these students consider online pre-seminar preparation unfavorable (Q10), prefer in-person frontal teaching over online videos (Q11) and find flipped classroom less helpful (Q12) which allows them less time to practice (Q15).

**Fig. 5:**
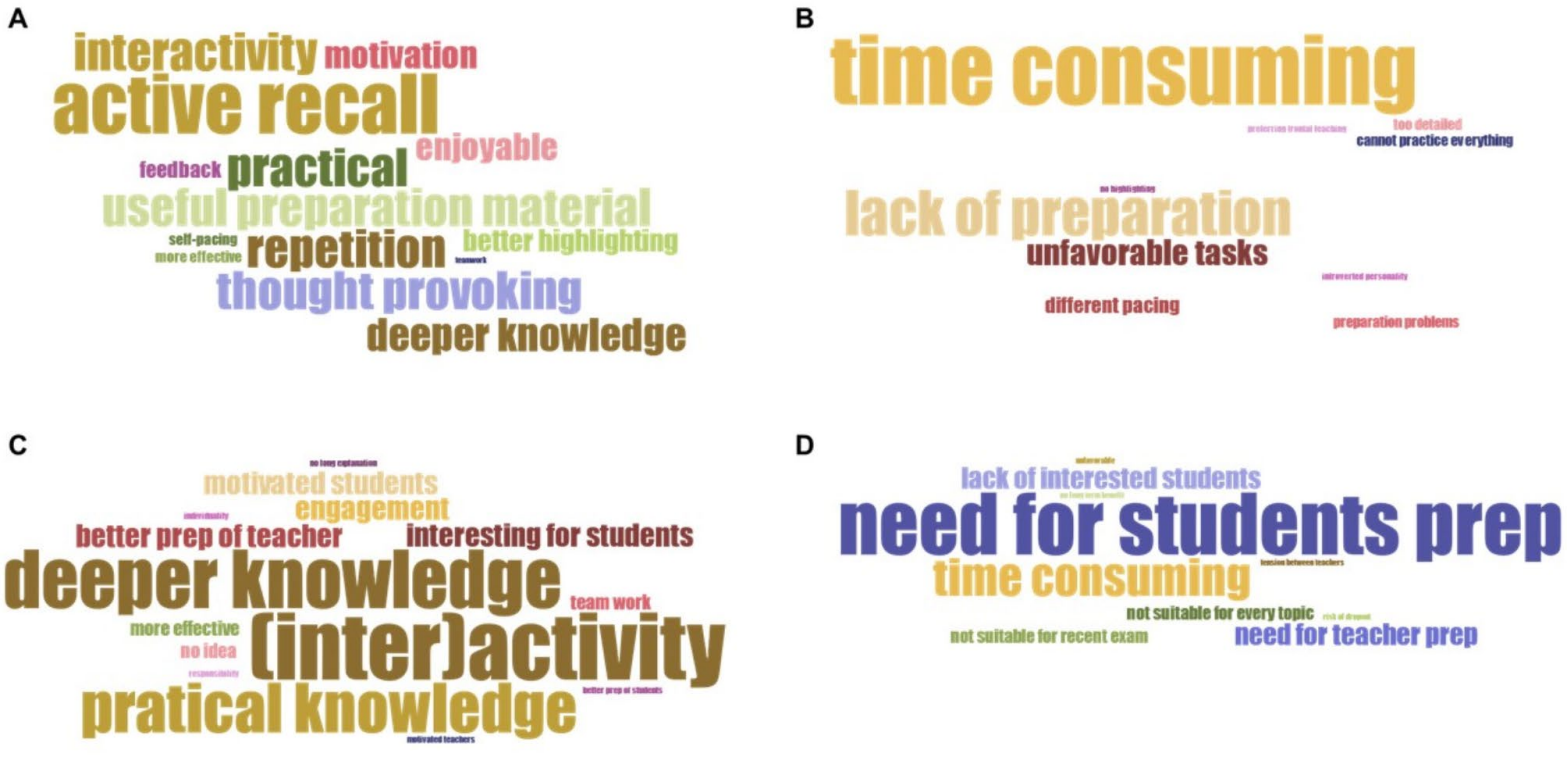
Word clouds generated by the keywords collected from students and teachers involved in our study. Advantages (**A**) and disadvantages (**B**) of flipped classroom approach according to the students. Advantages (**C**) and disadvantages (**D**) of the method according to the teachers. Word clouds were calculated and generated from responses from 44 students to open-ended questions

To further assess students’ opinion, we revised the feedback to open-ended questions, and we assigned a maximum of five keywords to each answer, then we visualized the keywords (Fig. 5). Students described the advantages of flipped classroom with the following keywords: active/better recall (10), interactivity (7), practical (7), useful preparation material (7) and thought provoking (7). As for disadvantages, students highlighted that they found flipped classroom time consuming (14), problematic if they are not prepared for the class (9), less favorable tasks (5) and interindividual differences in learning time (3).

**Table 2:** Spearman rank Rs. correlations between questions of students on flipped classroom approach

	FC No	Q1	Q2	Q3	Q4	Q5	Q6	Q7	Q8	Q9	Q10	Q11	Q12	Q13	Q14	Q15	Q16
*FC No: How many flipped classroom practices did you participate in during the two semesters?*	1,0	,24	-,20	-,02	-,14	,19	-,02	-,12	,12	-,18	,24	-,14	,30*	-,22	,06	-,19	,12
Q1: Flipped classroom practices are much more engaging than conventional classroom instruction.		1,0	-,50*	,10	-,23	,67*	,03	,06	,28	-,27	,26	-,29	,67*	-,16	,20	-,35*	,61*
Q2: I would not recommend flipped classroom practices to my classmates.			1,0	,21	,13	,02	,04	-,03	-,19	-,23	,12	-,08	,09	,10	-,25	-,09	,06
Q3: Flipped classroom practices give me more opportunities to communicate with my classmates.				1,0	,16	,13	,21	-,02	,07	-,41*	,37*	-,08	,17	,10	-,25	-,23	,27
Q4: I like to watch lesson material on video.					1,0	,16	-,09	-,06	-,07	,10	,17	-,15	-,01	,00	-,26	,17	,02
Q5: I would like the entire course to be held in a flipped classroom.						1,0	,02	,09	,14	-,16	,49*	-,32*	,66*	-,20	,16	-,15	,58*
Q6: I have to deal less time with the course material in a conventional classroom setting.							1,0	,12	-,27	,01	,10	,27	-,12	,20	-,16	,09	-,04
Q7: Social media (Facebook, YouTube, Twitter, Instagram, etc.) are not an important part of my learning process.								1,0	-,03	-,18	,16	-,24	,26	-,10	,03	-,12	-,11
Q8: I watch the video posted about the flipped classroom practice before class.									1,0	-,34*	,40*	-,04	,28	-,15	,09	-,01	,23
Q9: I am reluctant to solve assignments at my own pace.										1,0	-,42*	,30*	-,45*	,01	,08	,40*	-,22
Q10: I prefer to solve assignments online in Moodle during my preparation.											1,0	-,19	,49*	-,26	-,02	-,19	,32*
Q11: I prefer to participate live in a conventional seminar rather than viewing the material on video.												1,0	-,36*	,47*	,14	,35*	-,13
Q12: I feel that the flipped classroom helped me to learn the subject of pharmacology.													1,0	-,15	,12	-,51*	,56*
Q13: I am reluctant to assign myself the course material while taking the pharmacology course.														1,0	-,26	,28	-,08
Q14: I have no difficulty in assigning myself course material and keeping myself on schedule during the course.															1,0	,12	,15
Q15: The flipped classroom practice allows me less time to practice during class.																1,0	-,37*
Q16: I am more motivated when I can learn pharmacology in a flipped classroom																	1,0

*p < 0.05 was considered significant; *n* = 44

**Table 3:** Spearman rank Rs. correlations between questions of teachers on flipped classroom

	Exp	FC	Q1	Q2	Q3	Q4	Q5	Q6	Q7	Q8	Q9	Q10	Q11	Q12	Q13	Q14	Q15	Q16	Q17	Q18
*Exp: Overall teaching experience (years)*	1,00	-,04	-,53*	,03	,55*	-,07	,18	,13	,09	-,07	,07	,06	,02	-,02	,06	-,33	,01	0,22	0,17	0,22
*FC: Number of flipped classroom given by himself/herself*		1,00	,25	,10	,09	,13	,17	,25	-,20	-,31	,06	-,51*	,09	,22	-,52*	-,14	-,10	-0,36	0,29	0,35
Q1: In my work as a teacher, I regularly look up the details and background of each subject.			1,00	-,09	-,10	-,15	-,18	,00	,00	,34	,05	-,32	,03	,27	-,16	,18	-,22	-0,28	-0,13	-0,15
Q2: I try to incorporate the new pedagogical methods I learn about into my classes.				1,00	-,01	,62*	,46*	-,21	-,31	-,72*	-,60*	-,04	-,50*	-,58*	-,47*	-,53*	,62*	-0,56*	0,49*	0,34
Q3: I believe I give good quality classes.					1,00	-,18	-,04	,51	,08	,03	,10	-,28	-,20	,23	,10	,00	-,26	0,19	-0,16	-0,01
Q4: When a teacher changes from frontal teaching to a method that promotes active learning, the students’ performance improves.						1,00	,66*	-,51*	-,69*	-,76*	-,33	,32	-,24	-,55*	-,50*	-,59*	,64*	-0,48*	0,37	0,29
Q5: The flipped classroom gives my students a better understanding of the theoretical background of the course material.							1,00	-,50*	-,46	-,68*	-,14	,41	-,13	-,32	-,30	-,67*	,45	-0,35	0,42	0,62*
Q6: Not all my students (would) be successful with the flipped classroom method.								1,00	,53*	,32	,02	-,52*	-,14	,38	-,04	,21	-,44	0,11	-0,11	-0,10
Q7: The flipped classroom approach is (would be) a significant amount of extra work for me.									1,00	,49*	,04	-,08	-,05	,39	,43	,25	-,38	0,22	-0,20	-0,19
Q8: In my opinion, my student(s) would not prepare for my classes in advance, so active learning would not be achieved.										1,00	,32	-,08	,41	,61*	,52*	,65*	-,66*	0,48*	-0,52*	-0,51*
Q9: Students socialized in the current education system are unsuited to teaching in a different format than the frontal.											1,00	,29	,33	,20	,24	,39	-,58*	0,49*	-0,32	-0,17
Q10: I am disturbed when my students ask questions in class that are not related or only indirectly related to the subject matter.												1,00	,12	-,13	,37	-,10	,01	0,22	-0,09	-0,07
Q11: My students are expected to be able to master the material in detail independently with their mature learning methods.													1,00	,44	,29	,28	-,38	0,16	-0,20	-0,51*
Q12: The flipped classroom approach can frustrate students.														1,00	,48*	,48*	-,77*	0,20	-0,50*	-0,34
Q13: I would like to be the only one in charge of the class as the tutor, because over-involving students can make the class less effective.															1,00	,48*	-,37	0,57*	-0,64*	-0,37
Q14: If we share the practical material in video format, my status as a teacher becomes obsolete.																1,00	-,56*	0,39	-0,72*	-0,54*
Q15: The flipped classroom approach increases my students’ self-esteem.																	1,00	-0,37	0,33	0,47*
Q16: I prefer a frontal approach because my students’ behaviour and attention can be better controlled than in active learning.																		1,00	-0,31	-0,13
Q17: I am motivated when my students actively work and participate in my class.																			1,00	0,44
Q18: I can prepare more thoroughly before my flipped classroom type lessons about the background of the course material.																				1,00

*p < 0.05 was considered significant; *n* = 19

### Teachers raised concerns regarding student preparation and the workload on themselves

To introduce flipped classroom effectively, teacher perception needs to be evaluated as well. Previously, it was reported that flipped classroom might be perceived as a time-consuming method. Along the same the same line, general faculty resistance and preconceptions might complicate the introduction of the method. Thus, we investigated teacher perception by a questionnaire filled by teachers involved in either flipped or conventional teaching (Table 3); in addition, teachers who were not directly involved in these experiments, but having any experience with flipped classroom or conventional teaching were also asked to fill in the questionnaire.

In total, 19 teachers filled in the questionnaire. 10 teachers had previous experience with flipped classroom, majority of them held at least 2 flipped seminars, while 9 teachers had no experience. Interestingly, younger teachers with limited experience were represented extensively in our study as 13 teachers had only 5-year or less experience, while only 4 teachers had more than 10 years.

According to the questionnaire answers and subsequent Spearman correlation, teaching experience was negatively correlated with the need to look up the background of topics (Q1) and these teachers were also more confident on the quality of their classes (Q3). Teachers, who held more flipped seminars were less disturbed with students’ irrelevant questions (Q10) and being not in charge during the class (Q13).

Surprisingly, teachers who are more open about incorporating new teaching methods (Q2; answer 4–5 / agree-strongly agree; *n* = 13) were also optimistic on introducing flipped classroom including improving understanding and self-esteem of students (Q5, Q12, Q14, Q15), and student preparation and performance (Q4, Q8, Q9, Q17). In contrast, teachers who had concerns regarding student preparation (Q8 answer 4–5 / agree-strongly agree; *n* = 10) were less confident regarding flipped classroom efficiency (Q13), preferred conventional teaching (Q16), and they feel less motivated if students work during the class (Q17).

Large number of teachers irrespective of their preferred method supposed that flipped classroom is a significant amount of extra work (Q7; answer: 4–5 / agree-strongly agree; *n* = 14); correlation was found between the fear of extra workload and lack of student preparation (Q8).

For better understanding of teachers’ opinions, we assessed the open-ended questions, and we analyzed the answers as described above. Teachers collected the following keywords when describing the advantages of frontal teaching: suitable for every topic (12), less preparation for teachers (8), more effective (7), comfortable (6) and better control over the seminar (5). In contrast, advantages of flipped classroom were interactivity (8), deeper (7) and practical (7) knowledge, interesting (3) and motivating (3) for students. As for disadvantages, teachers mentioned that the need for student preparation (8) and being time consuming (5).

## Discussion

Flipped classroom is a well-known approach in teaching, which is used in a large variety of educational fields due to the positive effect on student engagement; however, less information has been available on its effects on student performance particularly on long term in pharmacology teaching for medical students. Here we demonstrated that flipped classroom significantly improved both short- and long-term performance of Hungarian medical students in pharmacology subject measured by classic multiple-choice tests. Furthermore, we also provided evidence that flipped classroom can be widely accepted among students. In contrast, teacher perceptions were rather mixed; excessive preparation as well as the presence of “faculty resistance” may complicate the implementation of this new method.

Flipped classroom is widely believed to facilitate learning process and has been tested for various subjects and fields including medical fields [4, 10, 15–18]. In our study, we successfully demonstrated that flipped classroom approach can be introduced to pharmacology teaching for Hungarian medical students. Our students generally performed better in the topics as we observed better performance in the test results of flipped classroom students immediately after the seminars. However, we need to highlight that we identified a topic from the four in which we did not detect this clear benefit; we hypothesize that low case number (i.e. large number of students missed the class due to other duties) and possibly the less suitable seminar plan led to the lack of improvement. Similarly, we did not evaluate the impact of the flipped classroom separately for students with different performance levels, including low performers, for whom it may be beneficial according to previous reports [19]; therefore, potential benefits might have been overlooked in our setting. In the future, we will revise the class plan and compare the results to clarify this potential discrepancy.

Flipped classroom approach may facilitate preparation prior to class, leading to better performance and engagement during the class. According to previous observations, many of the students spent time with preparation before the class [20]. In line with this finding, a large number of the students spent time with the preparation, which may partially explain the improvement. This hypothesis may also raise the concern of a possible disadvantage of flipped classroom which was mentioned by either our teachers, students, or other authors as lack of student preparation may compromise the benefits of flipped classroom or even increase the chance of dropout [9, 21]. Thus, we analyzed the impact of student preparation on this set of results. Surprisingly, students not prepared for the seminar may also have benefits from flipped approach as we found better performance of less prepared students at one topic compared to conventional groups. A possible explanation for this phenomenon is that flipped classroom may still offer a possibility to discuss and to learn the principles of the specific topics in a student-student or student-teacher setting as observed before [18, 22, 23]. However, we acknowledge that assessing the degree of student preparation presents several challenges, as both the speed and depth of preparation can vary significantly among individuals.

Long-term retention of knowledge is an important objective of pharmacology education. Previously, it was reported that students may recall important information even weeks after the flipped classroom [24]. However, contradictory data is also available on the long-term performance as decreased long-term knowledge retention was observed before [11, 12]. We examined the long-term knowledge retention in pharmacology teaching months after the original classes. In this study, we demonstrated that flipped classroom increased knowledge retention after two weeks in all topics in which we observed short-term benefits. Furthermore, we provided data on the better efficacy even after six months. This finding is more surprising knowing the fact that all the students successfully passed Pharmacology 1 exam two months before the long-term test, suggesting that most students spent time with learning the specific topics actively. Multiple factors may contribute to this observation. On one hand, we believe that flipped classroom students might have more experience with answering problem-oriented questions which were included in the long-term test compared to conventional students. On the other hand, students had personal experience of solving similar clinical cases which facilitated the recall of related knowledge. Nevertheless, the precise causes of surprising benefits should be clarified in the future.

As flipped classroom has been shown to be beneficial on student engagement [13], thus, we hypothesized that flipped classroom may improve the overall performance. In contrast to the topic-specific short- and long-term test, overall performance was not significantly improved based on the results of midterm tests or exams, which is in contrast to previous reports [25]. A few explanations may be behind our observation. Only two topics were flipped in our setting in each semester, while others (more than 20) were not; thus, low number of students passed their exam from the selected topics suggesting that overall performance could not show representative results regarding the selected topics. On the other hand, our recent assessment is an oral exam mainly concentrating on the theoretical background of each topic, while our short- and long-term tests contained various problem-oriented questions. This hypothesis may be confirmed by the subanalysis of midterm test questions; FC students performed slightly better in midterm questions related to FC topics even in case of the same overall performance; however, the low number of relevant midterm tests is a significant confounding factor and complicates making clear conclusions. Additionally, we acknowledge certain limitations due to the timing: potential interference between delayed and midterm tests may have influenced the results, which could significantly impact on our findings. This issue should be addressed in future research.

Flipped classroom might be more satisfying for undergraduate students, which was demonstrated by authors before [4]. Furthermore, flipped classroom has a series of positive pedagogical features as well, including flexibility or more interaction between students and teachers. In our study, these previous findings were further confirmed, as students involved and some of the teachers perceived flipped classroom more effective, interactive and flexible. In contrast to positive effects on student engagement and perception, some authors reported a strong preference for traditional teaching over alternative teaching methods [26]. Here, we found that students expressed resistance to the full adoption of flipped seminars, with the primary concern likely stemming from the demand for extensive pre-seminar preparation. Low interest in pre-seminar preparation has been reported before as well [27]. One plausible explanation for this opposition is the time-intensive nature of the preparatory materials. The use of alternative resources, such as interactive books, educational games, or articles with concise tasks, may alleviate this issue by reducing the preparation burden as reported before [28]. In this study, we provided students with various preparation materials, including downloadable handouts, specific scientific articles on certain topics and short videos accompanied by test questions to ensure immediate feedback. On the other hand, teachers implementing the flipped classroom model may encounter several challenges, particularly due to the perception that it means extra workload for teachers — an issue that has been raised as a concern in previous reports [17, 29, 30]. In our study, we observed a two-sided response among teachers. Those with prior experience in flipped classrooms were generally more receptive to implementing this approach, while others favored more traditional, conservative teaching methods. Interestingly, no significant age-related differences were noted, contrasting with previous findings that reported greater resistance to such innovations among older male faculty members [29]. As suggested before by others [31], teachers should shift their focus from class time to prioritizing students and their learning, which may take longer, particularly in countries where traditional teaching is favored over alternative methods. Notably, the evaluation of both student and teacher perceptions may be limited by the inherent subjectivity of their viewpoints and the lack of a detailed and fully validated analysis of interindividual differences. We suggest that integrating conventional frontal teaching with more interactive methods could help address this challenge in the future.

## Conclusions

In conclusion, the flipped classroom approach enhances topic-specific knowledge retention in pharmacology among medical students in both the short and long term, though it does not clearly improve overall performance, supporting its applicability in pharmacology education. Beyond its partial benefits on topic-specific performance, students favored this alternative teaching method over traditional settings. Nevertheless, student workload due to other educational duties and faculty resistance may pose significant challenges to the implementation of such alternative teaching methods.

## Electronic supplementary material

Below is the link to the electronic supplementary material.

Supplementary Material 1

## Data Availability

The datasets used and/or analyzed during the current study are available from the corresponding author on reasonable request.

## Abbreviations

FC: Flipped classroom
CON: Control
