# Supplementary material for: Implementing the flipped classroom model to enhance knowledge retention in pharmacology: a local case study at Semmelweis university
Source: BMC Med Educ. 2025 Feb 28;25:327. doi: 10.1186/s12909-025-06913-5 (PMC11871698; doi:10.1186/s12909-025-06913-5)
Supplement: Supplementary file 1 — Supplementary Material 1 [file 12909_2025_6913_MOESM1_ESM.docx]

**Appendices / Supplementary Material**

**Implementing the Flipped Classroom Model to Enhance Knowledge Retention in Pharmacology: A Local Case Study at Semmelweis University**

Running title: Flipped classroom in pharmacology teaching

Authors: Zsófia Onódi^1^, Pál Riba^1^, Péter Ferdinandy^1^, Anikó Görbe^1,*^, Zoltán V. Varga^1,†,*^

^1^Department of Pharmacology and Pharmacotherapy, Semmelweis University, Budapest, Hungary

^2^Center of Pharmacology and Drug Research & Development, Semmelweis University, Budapest, Hungary

^†^Corresponding author

Name: Zoltán V. Varga

address: H-1089 Budapest, Nagyvárad tér 4. Hungary

e-mail: varga.zoltan@semmelweis.hu

Tel: +36-1-210-4412

**Appendix 1**

**[TRANSLATED INTO ENGLISH FROM HUNGARIAN]**

**CONSENT TO THE STUDY ON EDUCATIONAL DEVELOPMENT**

**Effect of flipped classroom seminars on performance in pharmacology for third-year general medical students**

You are participating in a scientific research project led by *Dr. Anikó Görbe, Dr. Zsófia Gulyás-Onódi, Dr. Pál Riba and Dr. Zoltán Varga* (SE-ÁOK Department of Pharmacology and Pharmacotherapy).

The aim of this study is to explore the effects of the flipped classroom approach in the teaching of Pharmacology I-II. The study will explore changes in student performance, as well as student and teacher attitudes, expectations and subjective perceptions of the method. They provide insights into the effectiveness and applicability of the method.

**The study design:** Pharmacology I-II will be taught in the usual way to all students in most cases. The outcome requirements of the subject are unchanged. Over the two semesters, some groups will complete 2 of the 14 exercises (4 in total over the two semesters) using the so-called flipped classroom method.

**You are taking part in a conventional/flipped classroom method seminar.**

The duration of the seminar and the topics covered are the same as in conventional, i.e. traditional frontal-type seminars, but the pedagogical method is different.

**In the case of a conventional seminar**, the exercise is conducted in the usual way, through frontal explanations and class work, i.e. the student masters the material through the classroom explanations of the tutor by the end of the contact hour.

**In the case of a flipped classroom exercise**, the student prepares for the exercise at his/her own pace and preference, using the online materials provided in advance in Moodle. The exercise is interactive and involves problem-based tasks, usually in pairs or groups.

The resources uploaded to Moodle will be available to *all* students in the study, regardless of their pedagogical method, when preparing for the exam.

At the end of these exercises, each student will complete a *short multiple-choice test* on Moodle on the exercise material and provide a general, *fully anonymous student feedback* via Semmelweis University's Feedback system. The multiple-choice test measures the theoretical knowledge and competences acquired during the internship. Such tests may occur unannounced during the semester. **The results of the tests have no influence on the student's progress.** **They will not be included in any way in the mid-term, mid-semester or end-of-year assessment results.** Its sole purpose is to examine the effects of pedagogical methods on student performance.

There are no adverse consequences for physical or mental health, financial situation or any other factor in carrying out the above assessments.

**Participation in the survey is completely voluntary. You can stop participating or refuse to answer the questions at any time without giving any reason.**

There is no financial reward for participation in the research.

The results of the research will be published and presented at scientific conferences. These will be communicated orally or in writing, according to the wishes of the participants.

We keep *strictly confidential* all information (including test results and student opinions) that we collect as part of the research. Student reviews are completely *anonymous,* and the identity of the reviewers cannot be deciphered by the research managers. The data collected (name, email address, NEPTUN code and associated test results) and test results are not available to other Institute staff.

Content analysis will be performed on the data obtained from the survey, from which the identity of no participant can be established.

We attach the detailed information on data management (*Consent to data management*).

*I declare that I have been informed in detail about the data management and data security conditions of my participation in the research, that I agree to the conditions and that I consent to my participation. I also agree that any data collected about me during the study that cannot be used to identify me may be made available to other researchers. I reserve the right to withdraw from the study at any time during the study. In such a case, the data collected on me up to that point will be deleted.*

The SE-ÁOK Department of Pharmacology and Pharmacotherapy, as the data controller, will treat my above data (name, email address, NEPTUN code) confidentially and will not pass them on to any other data controller or processor.

You can find more information on the data processing regulations here:

<https://semmelweis.hu/jogigfoig/dokumentumtar/szabalyzattar/adatvedelmi-szabalyzat/>

*I have read and accept the document "Consent to data management". For matters not covered by this statement, the University Privacy Policy applies.*

yes, I accept no, I do not accept

*I also consent to the use of any data collected during the survey that cannot be used to identify me for research purposes and to its availability to other researchers. I declare that I have been informed in detail about the conditions and circumstances of my participation in the research, that I agree to the conditions and that I accept to participate.*

yes no

*I reserve the right to withdraw at any time during the investigation. In such a case, the data collected about me up to that point will be deleted.*

___________________, 20..... year …………….month……day

**__________________________**

**Student’s name:**

**Consent to data management**

Pursuant to Article 5(1)(a) of Act CXII of 2011 on Informational Self-Determination and Freedom of Information (Infotv.) and Articles 6(1)(a) and 9(2)(a) of the European General Data Protection Regulation (hereinafter GDPR), I consent to the processing of my data as detailed below.

**What can my data be used for?**

- For internal educational methodological developments

- For scientific presentations and publications (strictly anonymous)

**What data do I consent to?**

1. name and NEPTUN code for the duration of the study to link individual results

2. e-mail address for contact during the research period

**On what basis can my data be processed?**

My data can be processed because I have explicitly consented to this by means of this declaration.

**Who will be authorised to handle/process my data?**

Data controllers:

Department of Pharmacology and Pharmacotherapy, Semmelweis University

(XXX@semmelweis.hu; Tel: +36-1- XXX)

**How long will my data be processed?**

1 - 1.1 - 1.5 years

**I acknowledge that I have the following rights in relation to the processing of my data:**

1. the right to transparent information - I may request information about the processing of my personal data at any time;

2. the right of access to my personal data - I have the right to obtain information about my personal data processed by the controller;

3. the right to rectification, erasure of my data ("right to be forgotten") and restriction of processing - if my data is inaccurately processed by the controller, I can notify the controller and it will be corrected, erased, etc;

4. the controller is obliged to inform me of the identity of the recipients if it transfers my data to another party

5. the right to data portability - if I need it, they will provide me with my personal data;

6. the right to redress - if my rights are infringed, I can go to the Data Protection Officer, the National Authority for Data Protection and Freedom of Information or a court.

**Where can I go if I want to appeal or have questions about appeals?**

**Department of Pharmacology and Pharmacotherapy, SE ÁOK (XXX@semmelweis.hu; Tel: +36-1- XXX)**

**Semmelweis University Directorate General of Legal and Administrative Affairs**

Dr. Sára Trócsányi Data Protection Officer

Address for correspondence.

1085 Budapest, Baross utca 52.

Tel.: 06-1/XXX E-mail: XXX@semmelweis.hu

**To the National Authority for Data Protection and Freedom of Information**

1055 Budapest, Falk Miksa utca 9-11. Website: www.naih.hu

Phone: +36-1-XXX

**To the Court**

I can also take legal action in court, in Hungary I can choose to bring the action before the court of the place of residence or stay of the person concerned.

Date: __________________ (location) _______(year) ___________(month) _____(day)

________________________

**Signature**

Name: _________________________________________

NEPTUN code: _____________________

**Appendix 2**

**[TRANSLATED INTO ENGLISH FROM HUNGARIAN]**

**Students’ feedback questionnaire**

- *How many flipped classroom exercises have you participated in during the semester? 0 / 1 / 2 / 3 / 4*

**Likert scale questions (1 - strongly disagree, 5 - strongly agree)**

- *Flipped classroom exercises are much more engaging than traditional classroom instruction.*
- *I would not recommend flipped classroom exercises to my peers.*
- *Flipped classroom exercises give me more opportunities to communicate with my classmates.*
- *I like to watch the class material on video.*
- *I would like the whole course to be taught in a flipped classroom.*
- *I would have less to do with the course material in a traditional classroom setting.*
- *Social media (Facebook, YouTube, Twitter, Instagram, etc.) are not an important part of my learning process.*
- *I watch the video posted about the flipped classroom exercise before class.*
- *I am reluctant to solve assignments at my own pace.*
- *I am happy to solve exercises online in Moodle as part of my preparation.*
- *I prefer to attend a live traditional seminar rather than viewing the material on video.*
- *I feel that the flipped classroom helped me to learn the subject of pharmacology.*
- *I am reluctant to assign myself the course material when taking a pharmacology course.*
- *I have no difficulty in assigning the course material during the course and sticking to the schedule.*
- *Flipped classroom practice allows me less time to practice during class.*
- *I am much more motivated when I can learn pharmacology in a flipped classroom.*

**Open-ended questions:**

- *What benefits do you see for flipped classroom practice?*
- *What disadvantages do you see of flipped classroom practice?*
- *Would you learn any other subject using the flipped classroom method? If so, why? If not, why?*
- *In what ways do you think flipped classroom practices could be improved?*
- *Share any other comments or suggestions you have:*

**Appendix 3**

**[TRANSLATED INTO ENGLISH FROM HUNGARIAN]**

**Teachers’ feedback questionnaire**

- *Are you participating as a teacher in the FC project for the academic year 2023/24? yes, I’m a teacher of a conventional groups / yes, I’m a teacher of a FC groups / no*
- *How many years of independent teaching experience do you have? Less than 2 years / 2-5 years / 5-10 years / 10-20 years / more than 20 years*
- *Do you have regular clinical pharmacology practice? (Regularly = at least 1 time per month) yes, using team-based learning methods / yes, holding frontal presentations / rarely / no*
- *Have you taught using flipped classroom methods in the last two years? If yes, when? yes, this year / yes, last year / no / other (I’m specifying)*
- *How many flipped classroom exercises have you done in total? (1 = one full exercise) 1 / 2-3 / 4 or more / never*
- *Which form of teaching do you currently prefer? Exclusively frontal / mainly frontal with occasional flipped / exclusively flipped classroom / hybrid education dividing seminar into frontal and interactive part*

**Likert scale questions (1 - strongly disagree, 5 - strongly agree)**

- *In my work as an instructor, I regularly check the details and background of each course.*
- *In addition to the course material, I prepare a detailed lesson plan in preparation for the lessons in order to better manage my time.*
- *I regularly attend training courses and level lectures on the subject matter.*
- *I am concerned that I cannot answer my students' questions accurately.*
- *I attend training courses on pedagogical methods.*
- *I try to incorporate the pedagogical methods I learn about into my lessons.*
- *The current teaching structure supports the integration of new pedagogical methods.*
- *It is very important to me that my students understand the material I teach.*
- *I always start the lesson on time.*
- *I think it's important for me to stimulate my students' interest in the subject matter.*
- *The effectiveness of teaching depends first and foremost on me, the teacher.*
- *I like my students to ask questions.*
- *In my opinion, students should not be allowed to question why certain parts of the curriculum need to be learnt.*
- *I pay attention to the physical and psychological needs of my students during my lessons so that they do not interfere with the learning process.*
- *Sometimes I have noticed that a student is not in a fit state to learn because of a physical/psychological problem.*
- *I also provide extra material for my students who are interested, to help them prepare.*
- *I get frustrated when my students' performance is below expectations.*
- *I believe I teach good quality classes.*
- *The oral examination is the most practical form of assessment for the pharmacology subject.*
- *During the oral exam I can easily judge whether the student can apply what he/she has learned.*
- *When an instructor replaces frontal teaching with a method that promotes active learning, student performance improves.*
- *If I use gamification to teach the material, I can become frivolous in the eyes of my students and colleagues.*
- *The flipped classroom gives my students a better understanding of the theoretical background of the course material.*
- *I consider it essential to develop my students' ability to work in teams in my own classes.*
- *Not all my students (would) be successful with the flipped classroom method.*
- *The flipped classroom approach means(s) a significant amount of extra work for me.*
- *In my opinion, my student(s) would not prepare in advance for my classes, so active learning would not be achieved.*
- *Students socialized in the current education system are unsuited to teaching in a different format than the frontal.*
- *I am disturbed when my students ask questions in class that are not related or only indirectly related to the subject matter.*
- *My students are expected to be able to master the material in detail independently with their mature learning methods.*
- *The flipped classroom approach can frustrate students.*
- *As the tutor, I would like to lead the class exclusively, as over-involvement of students can undermine the effectiveness of the class.*
- *If we share the practical material in video format, my status as an instructor becomes redundant.*
- *The flipped classroom approach increases my students' self-esteem.*
- *I believe that the flipped classroom gives students more responsibility for their own progress.*
- *I prefer the frontal approach because my students' behavior and attention can be better controlled than in active learning.*
- *I am motivated when my students are actively working and participating in my lessons.*
- *I can prepare more thoroughly before my flipped classroom type lessons about the background of the course material.*

**Open-ended questions:**

- *For me, the advantages of frontal teaching:*
- *For me, the advantages of flipped classroom:*
- *I would not choose the flipped classroom approach because...*
- *As an educator, it/those would help me most in my work if:*
- *Any other comments I would like to share:*

1. **Supplementary tables**

|  | **CON1** | **CON2** | **CON3** | **CON4** | **CON5** | **FC1** | **FC2** | **FC3** | **FC4** | **FC5** |
| --- | --- | --- | --- | --- | --- | --- | --- | --- | --- | --- |
| Assigned teacher(s) | T1 | T2 | T3 | T4 | T5 / T10 | T6 | T7 | T9 / T6 | T8 | T9 |
| Male | 7 | 6 | 6 | 6 | 9 | 7 | 7 | 8 | 8 | 5 |
| Female | 10 | 9 | 13 | 12 | 7 | 9 | 10 | 8 | 7 | 7 |
| Total (male) |  |  |  |  | **34** |  |  |  |  | **35** |
| Total (female) |  |  |  |  | **51** |  |  |  |  | **41** |
|  |  |  |  |  |  |  |  |  |  |  |

**Supplementary table 1 – Summary of student demographics in the control (CON) and flipped classroom (FC) groups.** Student characteristics include total number, gender distribution, and group assignment method. Abbrevations: T – teacher.

|  | **CON** | **FC** |
| --- | --- | --- |
| Median age | 31,5 | 31 |
| Gender (M/F) | 5 / 1 | 2 / 2 |
| Number of teachers with less than 2-year teaching experience | 2 | 2 |
| Number of teachers with more than 5-year teaching experience | 2 | 2 |

**Supplementary table 2 – Summary of teacher demographics in the control (CON) and flipped classroom (FC) groups.** Teacher characteristics include median age, gender distribution, and teaching experience. Abbreviations: M – male, F – female.

|  | **Traditional (frontal) teaching** | **Flipped classroom** |
| --- | --- | --- |
| **Group size** | 15-25 students | |
| **Available online material** | - presentation slide show - additional material (such as relevant scientific articles) | - presentation slide show - short video presentations supplemented with test questions - additional material (such as relevant scientific articles) |
| **Seminar structure** | Frontal presentation | Blended learning |
| **Applied techniques during the seminar** | - slide presentation(s) and note preparation by teachers using whiteboard - short question and answer session when demanded | - case- and problem-based discussion in small groups (3-4 students) - short frontal explanation on complex pharmacological problems |
| **Assessment of performance** | - early, delayed and long-term tests after the seminars - midterm tests (formative assessment) - exam and final exam (summative assessment) | |
| **Assessment of student perception** | - immediate official questionnaire by the university | - immediate official questionnaire by the university - Likert scale and open ended question-based questionnaire to assess perception on flipped seminars |

**Supplementary table 3 – Differences and similarities between the structure of traditional and flipped seminars.**

|  | **Early test results** | | | | | | | | | | | | | | | | | | | |
| --- | --- | --- | --- | --- | --- | --- | --- | --- | --- | --- | --- | --- | --- | --- | --- | --- | --- | --- | --- | --- |
|  | **Topic 1** | | | | | **Topic 2** | | | | | **Topic 3** | | | | | **Topic 4** | | | | |
|  | **Mean** | **Median** | **Range** | **Number of participants** | **P** | **Mean** | **Median** | **Range** | **Number of participants** | **P** | **Mean** | **Median** | **Range** | **Number of participants** | **P** | **Mean** | **Median** | **Range** | **Number of participants** | **P** |
| **CON** | 44.42 | 47 | 7-80 | 72 | 0.0003 | 45.47 | 47 | 13-80 | 62 | 0.1005 | 66.95 | 67 | 27-100 | 65 | 0.0404 | 38.09 | 40 | 7-80 | 55 | 0.0006 |
| **FC** | 55.66 | 53 | 20-100 | 65 |  | 50.60 | 47 | 13-80 | 58 |  | 74.85 | 73 | 33-100 | 61 |  | 50.94 | 47 | 13-87 | 52 |  |

| **Early test results (by groups)** | | | | | | | | | | | | | | | | | | | | | | |
| --- | --- | --- | --- | --- | --- | --- | --- | --- | --- | --- | --- | --- | --- | --- | --- | --- | --- | --- | --- | --- | --- | --- |
|  | **Topic 1** | | | | | | | | | |  |  | **Topic 3** | | | | | | | | | |
|  | **CON** | | | | | **FC** | | | | |  |  | **CON** | | | | | **FC** | | | | |
| **Number of values** | 13 | 14 | 16 | 15 | 14 | 15 | 16 | 10 | 15 | 9 |  | **Number of values** | 10 | 14 | 14 | 13 | 14 | 11 | 14 | 14 | 11 | 11 |
|  |  |  |  |  |  |  |  |  |  |  |  |  |  |  |  |  |  |  |  |  |  |  |
| **Minimum** | 13 | 20 | 20 | 27 | 7 | 40 | 33 | 20 | 27 | 27 |  | **Minimum** | 60 | 27 | 33 | 53 | 40 | 53 | 60 | 47 | 60 | 33 |
| **25% Percentile** | 43.5 | 27 | 40 | 40 | 20 | 47 | 47 | 38.25 | 40 | 43.5 |  | **25% Percentile** | 71.5 | 33 | 51.5 | 70 | 40 | 60 | 67 | 65.25 | 60 | 60 |
| **Median** | 47 | 40 | 50 | 53 | 27 | 60 | 47 | 57 | 53 | 53 |  | **Median** | 83.5 | 43.5 | 60 | 80 | 67 | 67 | 83.5 | 73 | 87 | 73 |
| **75% Percentile** | 56.5 | 50.25 | 60 | 67 | 47 | 80 | 65.25 | 80 | 60 | 67 |  | **75% Percentile** | 93 | 68.5 | 73 | 87 | 88.5 | 80 | 93 | 87 | 93 | 80 |
| **Maximum** | 73 | 73 | 73 | 80 | 47 | 87 | 87 | 100 | 73 | 73 |  | **Maximum** | 93 | 93 | 87 | 93 | 100 | 87 | 100 | 93 | 100 | 87 |
| **Range** | 60 | 53 | 53 | 53 | 40 | 47 | 54 | 80 | 46 | 46 |  | **Range** | 33 | 66 | 54 | 40 | 60 | 34 | 40 | 46 | 40 | 54 |
|  |  |  |  |  |  |  |  |  |  |  |  |  |  |  |  |  |  |  |  |  |  |  |
| **Mean** | 48.23 | 42 | 48.69 | 51.2 | 31.14 | 61.8 | 54.81 | 58.7 | 50.2 | 52.67 |  | **Mean** | 80.6 | 52.36 | 61.86 | 78.46 | 66.21 | 69.73 | 80.86 | 73.86 | 79.91 | 68.55 |
| **Std. Deviation** | 15 | 16.4 | 16.03 | 16.28 | 12.75 | 16.68 | 14.25 | 25.1 | 12.76 | 14.61 |  | **Std. Deviation** | 11.87 | 21.41 | 16.08 | 12.58 | 21.45 | 11.77 | 13.92 | 12.9 | 15.18 | 17.24 |
| **Std. Error of Mean** | 4.159 | 4.383 | 4.008 | 4.203 | 3.406 | 4.306 | 3.563 | 7.936 | 3.295 | 4.871 |  | **Std. Error of Mean** | 3.754 | 5.723 | 4.298 | 3.489 | 5.732 | 3.55 | 3.721 | 3.446 | 4.576 | 5.197 |
|  |  |  |  |  |  |  |  |  |  |  |  |  |  |  |  |  |  |  |  |  |  |  |
| **Subcolumn analysis (Chi-square)** | Yes. P=0.0452 | | | | | | | | | |  | **Subcolumn analysis (Chi-square)** | Yes. P=0.0007 | | | | | | | | | |
|  | **Topic 2** | | | | | | | | | |  |  | **Topic 4** | | | | | | | | | |
|  | **CON** | | | | | **FC** | | | | |  |  | **CON** | | | | | **FC** | | | | |
| **Number of values** | 12 | 15 | 16 | 15 | 4 | 11 | 12 | 14 | 12 | 9 |  | **Number of values** | 6 | 14 | 9 | 12 | 14 | 11 | 9 | 11 | 12 | 9 |
|  |  |  |  |  |  |  |  |  |  |  |  |  |  |  |  |  |  |  |  |  |  |  |
| **Minimum** | 40 | 20 | 20 | 13 | 20 | 33 | 20 | 13 | 27 | 27 |  | **Minimum** | 40 | 7 | 20 | 13 | 7 | 20 | 20 | 13 | 33 | 27 |
| **25% Percentile** | 47 | 27 | 40 | 33 | 23.25 | 47 | 33 | 36.75 | 41.75 | 36.5 |  | **25% Percentile** | 45.25 | 18.25 | 33.5 | 33 | 18.25 | 40 | 43.5 | 40 | 40 | 40 |
| **Median** | 56.5 | 33 | 56.5 | 47 | 36.5 | 67 | 40 | 47 | 60 | 53 |  | **Median** | 53 | 27 | 47 | 43.5 | 27 | 47 | 47 | 53 | 53 | 40 |
| **75% Percentile** | 65.25 | 40 | 65.25 | 53 | 64.75 | 67 | 51.5 | 56.5 | 67 | 70 |  | **75% Percentile** | 54.75 | 47 | 60 | 56.75 | 41.75 | 60 | 63.5 | 67 | 67 | 53 |
| **Maximum** | 73 | 53 | 73 | 80 | 73 | 80 | 67 | 80 | 73 | 80 |  | **Maximum** | 60 | 53 | 80 | 73 | 73 | 73 | 67 | 100 | 80 | 67 |
| **Range** | 33 | 33 | 53 | 67 | 53 | 47 | 47 | 67 | 46 | 53 |  | **Range** | 20 | 46 | 60 | 60 | 66 | 53 | 47 | 87 | 47 | 40 |
|  |  |  |  |  |  |  |  |  |  |  |  |  |  |  |  |  |  |  |  |  |  |  |
| **Mean** | 56.67 | 33.73 | 51.31 | 43.07 | 41.5 | 59.55 | 40.5 | 46.21 | 55.58 | 53.33 |  | **Mean** | 51 | 29.14 | 47.56 | 43.83 | 30.5 | 49.73 | 49.78 | 54.64 | 53.83 | 45.22 |
| **Std. Deviation** | 10.7 | 8.795 | 16.72 | 17.5 | 22.58 | 14.14 | 13.52 | 18.93 | 14.49 | 19.15 |  | **Std. Deviation** | 6.782 | 15.13 | 18.37 | 18.53 | 19.25 | 15.89 | 14.64 | 25.35 | 14.94 | 11.4 |
| **Std. Error of Mean** | 3.088 | 2.271 | 4.18 | 4.518 | 11.29 | 4.263 | 3.903 | 5.06 | 4.182 | 6.384 |  | **Std. Error of Mean** | 2.769 | 4.045 | 6.124 | 5.348 | 5.145 | 4.79 | 4.878 | 7.644 | 4.313 | 3.8 |
|  |  |  |  |  |  |  |  |  |  |  |  |  |  |  |  |  |  |  |  |  |  |  |
| **Subcolumn analysis (Chi-square)** | Yes. P=0.0016 | | | | | | | | | |  | **Subcolumn analysis (Chi-square)** | No. P>0.05. | | | | | | | | | |

**Supplementary table 4 –** **The impact of flipped classroom on student performance in short-term (table format). The results of 15-question tests performed immediately after seminars. *P<0.05 CON vs FC, Mann-Whitney test. Subgroup analysis was performed with Chi-square test. Abbreviations: CON – conventional, FC – flipped classroom, Std – standard.**

|  | **Impact of preparation on the outcomes of flipped classroom** | | | | | | | | | | | | | | | | | | | |
| --- | --- | --- | --- | --- | --- | --- | --- | --- | --- | --- | --- | --- | --- | --- | --- | --- | --- | --- | --- | --- |
|  | **Topic 1** | | | | | **Topic 2** | | | | | **Topic 3** | | | | | **Topic 4** | | | | |
|  | **Mean** | **Median** | **Range** | **N** | **P** | **Mean** | **Median** | **Range** | **N** | **P** | **Mean** | **Median** | **Range** | **N** | **P** | **Mean** | **Median** | **Range** | **N** | **P** |
| **Not prepared** | 53.37 | 53 | 20-87 | 19 | 0.6614 | 38.81 | 40 | 13-60 | 16 | 0.0015 | 66.33 | 60 | 33-93 | 21 | 0.0018 | 50.71 | 47 | 27-67 | 17 | 0.9107 |
| **Prepared** | 56.61 | 53 | 27-100 | 46 |  | 55.10 | 56.50 | 20-80 | 42 |  | 79.33 | 80 | 60-100 | 40 |  | 51.06 | 47 | 13-100 | 35 |  |

**Supplementary table 5 - The impact of preparation prior flipped classroom on student performance in short-term (table format). The results of 15-question tests of students participating at flipped seminar with or without significant preparation were compared to the conventional classroom results.** ***P<0.05 CON vs FC, Mann-Whitney test.** **Abbreviations: N – number of participants.**

|  | **Delayed test results** | | | | | | | | | | | | | | | | | | | |
| --- | --- | --- | --- | --- | --- | --- | --- | --- | --- | --- | --- | --- | --- | --- | --- | --- | --- | --- | --- | --- |
|  | **Topic 1** | | | | | **Topic 2** | | | | | **Topic 3** | | | | | **Topic 4** | | | | |
|  | **Mean** | **Median** | **Range** | **N** | **P** | **Mean** | **Median** | **Range** | **N** | **P** | **Mean** | **Median** | **Range** | **N** | **P** | **Mean** | **Median** | **Range** | **N** | **P** |
| CON | 32.84 | 33 | 13-73 | 55 | 0.0323 | 55.03 | 60 | 7-87 | 34 | 0.1968 | 43.75 | 40 | 13-87 | 62 | 0.0175 | 27.86 | 27 | 0-80 | 49 | 0.0343 |
| FC | 37.72 | 40 | 7-67 | 58 |  | 61.56 | 67 | 13-93 | 39 |  | 52.84 | 53 | 13-87 | 50 |  | 34.02 | 33 | 7-73 | 45 |  |

| **Delayed test results (by groups)** | | | | | | | | | | | | | | | | | | | | | | |
| --- | --- | --- | --- | --- | --- | --- | --- | --- | --- | --- | --- | --- | --- | --- | --- | --- | --- | --- | --- | --- | --- | --- |
|  | **Topic 1** | | | | | | | | | |  |  | **Topic 3** | | | | | | | | | |
|  | **CON** | | | | | **FC** | | | | |  |  | **CON** | | | | | **FC** | | | | |
| **Number of values** | 6 | 12 | 14 | 13 | 10 | 14 | 13 | 9 | 13 | 9 |  | **Number of values** | 3 | 12 | 15 | 16 | 16 | 5 | 10 | 14 | 12 | 9 |
|  |  |  |  |  |  |  |  |  |  |  |  |  |  |  |  |  |  |  |  |  |  |  |
| **Minimum** | 13 | 13 | 13 | 13 | 13 | 13 | 13 | 20 | 7 | 20 |  | **Minimum** | 13 | 20 | 13 | 13 | 20 | 33 | 13 | 27 | 20 | 27 |
| **25% Percentile** | 23.5 | 20 | 18.25 | 27 | 20 | 33 | 27 | 27 | 30 | 23.5 |  | **25% Percentile** | 13 | 27 | 40 | 40 | 33 | 36.5 | 38.5 | 38.25 | 27 | 43.5 |
| **Median** | 36.5 | 23.5 | 33 | 33 | 27 | 40 | 33 | 40 | 33 | 33 |  | **Median** | 40 | 33 | 47 | 50 | 40 | 53 | 63.5 | 47 | 40 | 60 |
| **75% Percentile** | 48.5 | 38.25 | 41.75 | 57 | 41.75 | 48.5 | 50 | 47 | 50 | 43.5 |  | **75% Percentile** | 73 | 45.25 | 53 | 65.25 | 45.25 | 70 | 80 | 63.25 | 71.5 | 70 |
| **Maximum** | 53 | 67 | 53 | 73 | 47 | 67 | 67 | 53 | 60 | 47 |  | **Maximum** | 73 | 47 | 73 | 87 | 60 | 73 | 87 | 87 | 87 | 80 |
| **Range** | 40 | 54 | 40 | 60 | 34 | 54 | 54 | 33 | 53 | 27 |  | **Range** | 60 | 27 | 60 | 74 | 40 | 40 | 74 | 60 | 67 | 53 |
|  |  |  |  |  |  |  |  |  |  |  |  |  |  |  |  |  |  |  |  |  |  |  |
| **Mean** | 35.5 | 28.83 | 30.86 | 40.08 | 29.4 | 40.93 | 37.46 | 37.11 | 37.92 | 33.44 |  | **Mean** | 42 | 35.08 | 46.2 | 51.63 | 40.38 | 53.2 | 57.4 | 51.5 | 47.25 | 57.11 |
| **Std. Deviation** | 14.45 | 15.25 | 13.59 | 19.1 | 11.97 | 12.86 | 15.32 | 11.06 | 14.68 | 10.6 |  | **Std. Deviation** | 30.05 | 9.1 | 14.55 | 18.38 | 11.58 | 17.06 | 26.13 | 18.99 | 24.03 | 16.95 |
| **Std. Error of Mean** | 5.898 | 4.402 | 3.631 | 5.298 | 3.787 | 3.436 | 4.248 | 3.687 | 4.071 | 3.532 |  | **Std. Error of Mean** | 17.35 | 2.627 | 3.756 | 4.594 | 2.895 | 7.632 | 8.264 | 5.075 | 6.937 | 5.651 |
|  |  |  |  |  |  |  |  |  |  |  |  |  |  |  |  |  |  |  |  |  |  |  |
| **Subcolumn analysis (Chi-square)** | No. P>0.05. | | | | | | | | | |  | **Subcolumn analysis (Chi-square)** | No. P>0.05. | | | | | | | | | |
|  | **Topic 2** | | | | | | | | | |  |  | **Topic 4** | | | | | | | | | |
|  | **CON** | | | | | **FC** | | | | |  |  | **CON** | | | | | **FC** | | | | |
| **Number of values** | 6 | 8 | 8 | 12 | 2 | 7 | 10 | 4 | 10 | 8 |  | **Number of values** | 6 | 14 | 7 | 8 | 14 | 9 | 6 | 11 | 13 | 6 |
|  |  |  |  |  |  |  |  |  |  |  |  |  |  |  |  |  |  |  |  |  |  |  |
| **Minimum** | 27 | 7 | 20 | 40 | 13 | 40 | 13 | 73 | 27 | 13 |  | **Minimum** | 27 | 7 | 7 | 20 | 0 | 33 | 20 | 13 | 7 | 20 |
| **25% Percentile** | 46.5 | 18 | 23.25 | 54.75 | 13 | 53 | 38.5 | 73 | 45.25 | 53 |  | **25% Percentile** | 27 | 18.25 | 13 | 28.5 | 11.5 | 33 | 25.25 | 20 | 23.5 | 29.75 |
| **Median** | 60 | 60 | 50 | 73 | 16.5 | 67 | 63.5 | 73 | 47 | 63.5 |  | **Median** | 30 | 33 | 20 | 33 | 20 | 40 | 40 | 27 | 33 | 36.5 |
| **75% Percentile** | 63.25 | 65.25 | 60 | 73 | 20 | 87 | 80 | 88 | 70.25 | 83.5 |  | **75% Percentile** | 41.75 | 41.75 | 20 | 49.75 | 33 | 53.5 | 53 | 27 | 36.5 | 40 |
| **Maximum** | 73 | 73 | 67 | 87 | 20 | 87 | 87 | 93 | 87 | 93 |  | **Maximum** | 47 | 47 | 53 | 80 | 40 | 60 | 53 | 73 | 53 | 40 |
| **Range** | 46 | 66 | 47 | 47 | 7 | 47 | 74 | 20 | 60 | 80 |  | **Range** | 20 | 40 | 46 | 60 | 40 | 27 | 33 | 60 | 46 | 20 |
|  |  |  |  |  |  |  |  |  |  |  |  |  |  |  |  |  |  |  |  |  |  |  |
| **Mean** | 55.5 | 46.63 | 45 | 67.08 | 16.5 | 67.71 | 57.4 | 78 | 54.2 | 62.38 |  | **Mean** | 33.5 | 28.57 | 20.86 | 39.88 | 21.36 | 42.89 | 38.83 | 27.91 | 30.69 | 34.33 |
| **Std. Deviation** | 15.4 | 25.45 | 18.5 | 13.21 | 4.95 | 17.11 | 26.13 | 10 | 18.43 | 24.78 |  | **Std. Deviation** | 8.385 | 14.2 | 15 | 18.84 | 12.2 | 11.29 | 14.12 | 15.92 | 11.24 | 7.815 |
| **Std. Error of Mean** | 6.286 | 8.998 | 6.541 | 3.813 | 3.5 | 6.469 | 8.264 | 5 | 5.827 | 8.761 |  | **Std. Error of Mean** | 3.423 | 3.795 | 5.671 | 6.661 | 3.261 | 3.762 | 5.764 | 4.8 | 3.116 | 3.19 |
|  |  |  |  |  |  |  |  |  |  |  |  |  |  |  |  |  |  |  |  |  |  |  |
| **Subcolumn analysis (Chi-square)** | No. P>0.05. | | | | | | | | | |  | **Subcolumn analysis (Chi-square)** | Yes. P=0.0372 | | | | | | | | | |

**Supplementary table 6 - The impact of flipped classroom on student performance (table format). The results of 15-question tests performed two weeks after seminars. *P CON vs FC, Mann-Whitney test. Subgroup analysis was performed with Chi-square test. Abbreviations: CON – conventional, FC – flipped classroom, Std – standard.**

|  | **Long-term test results** | | | | |
| --- | --- | --- | --- | --- | --- |
|  | **Topic 1-2** | | | | |
|  | **Mean** | **Median** | **Range** | **Number of participants** | **P** |
| **CON** | 39.13 | 40 | 13-87 | 63 | 0.0106 |
| **FC** | 48.14 | 47 | 13-87 | 59 |  |

|  |  | | | | | | | | | |
| --- | --- | --- | --- | --- | --- | --- | --- | --- | --- | --- |
|  | **Long-term test (FC #1-2)** | | | | | | | | | |
|  | **CON** | | | | | **FC** | | | | |
| **Number of values** | 11 | 13 | 14 | 13 | 12 | 12 | 10 | 14 | 12 | 11 |
|  |  |  |  |  |  |  |  |  |  |  |
| **Minimum** | 20 | 13 | 20 | 20 | 27 | 27 | 33 | 20 | 20 | 13 |
| **25% Percentile** | 20 | 20 | 33 | 27 | 33 | 33 | 38.25 | 27 | 28.5 | 20 |
| **Median** | 33 | 40 | 47 | 33 | 36.5 | 53.5 | 53 | 33 | 47 | 53 |
| **75% Percentile** | 40 | 53 | 54.75 | 50 | 45.25 | 71.5 | 68.5 | 54.75 | 67 | 67 |
| **Maximum** | 47 | 60 | 73 | 87 | 60 | 87 | 80 | 80 | 73 | 80 |
| **Range** | 27 | 47 | 53 | 67 | 33 | 60 | 47 | 60 | 53 | 67 |
|  |  |  |  |  |  |  |  |  |  |  |
| **Mean** | 32.18 | 37.85 | 44.21 | 40.54 | 39.42 | 51.67 | 53.9 | 41.36 | 47.33 | 48.55 |
| **Std. Deviation** | 10.3 | 17.73 | 15.37 | 19.23 | 11.2 | 20.93 | 16.23 | 18.84 | 19.42 | 22.25 |
| **Std. Error of Mean** | 3.107 | 4.917 | 4.109 | 5.333 | 3.232 | 6.041 | 5.133 | 5.036 | 5.605 | 6.707 |
|  |  |  |  |  |  |  |  |  |  |  |
| **Subcolumn analysis (Chi-square)** | No. P>0.05. | | | | | | | | | |

**Supplementary table 7 - The impact of flipped classroom on student performance in long term (table format). The result of a 15-question test performed six months after the first two seminars (topic of FC #1 and 2). *P CON vs FC, Mann-Whitney test. Subgroup analysis was performed with Chi-square test. Abbreviations: CON – conventional, FC – flipped classroom, Std – standard.**

1. **Supplementary Figures and figure legends**

**
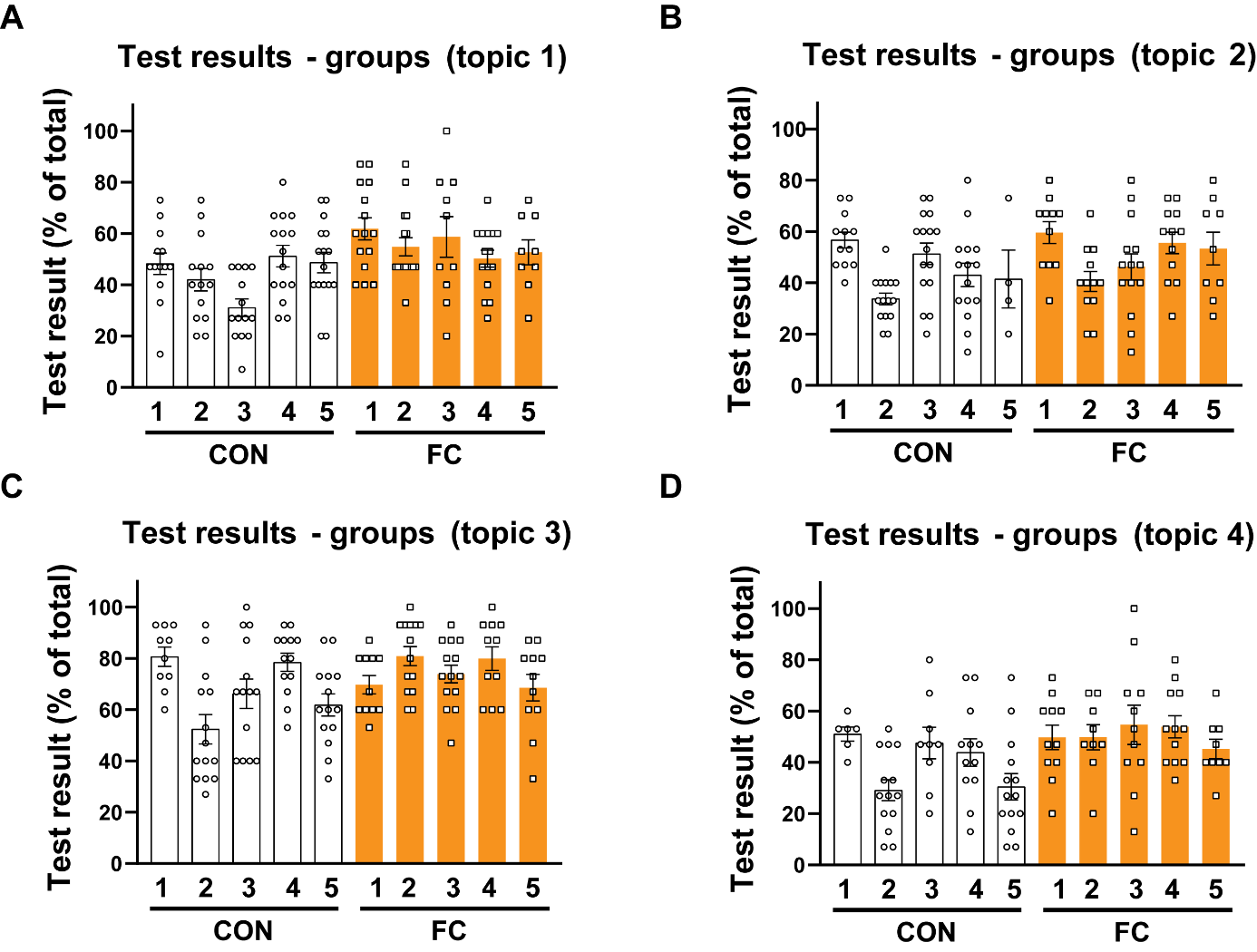
**

**Supplementary Figure 1 – The impact of flipped classroom on student performance in short-term. Subgroup analysis was performed with Chi-square test. (see Supplementary Table 4)**

**
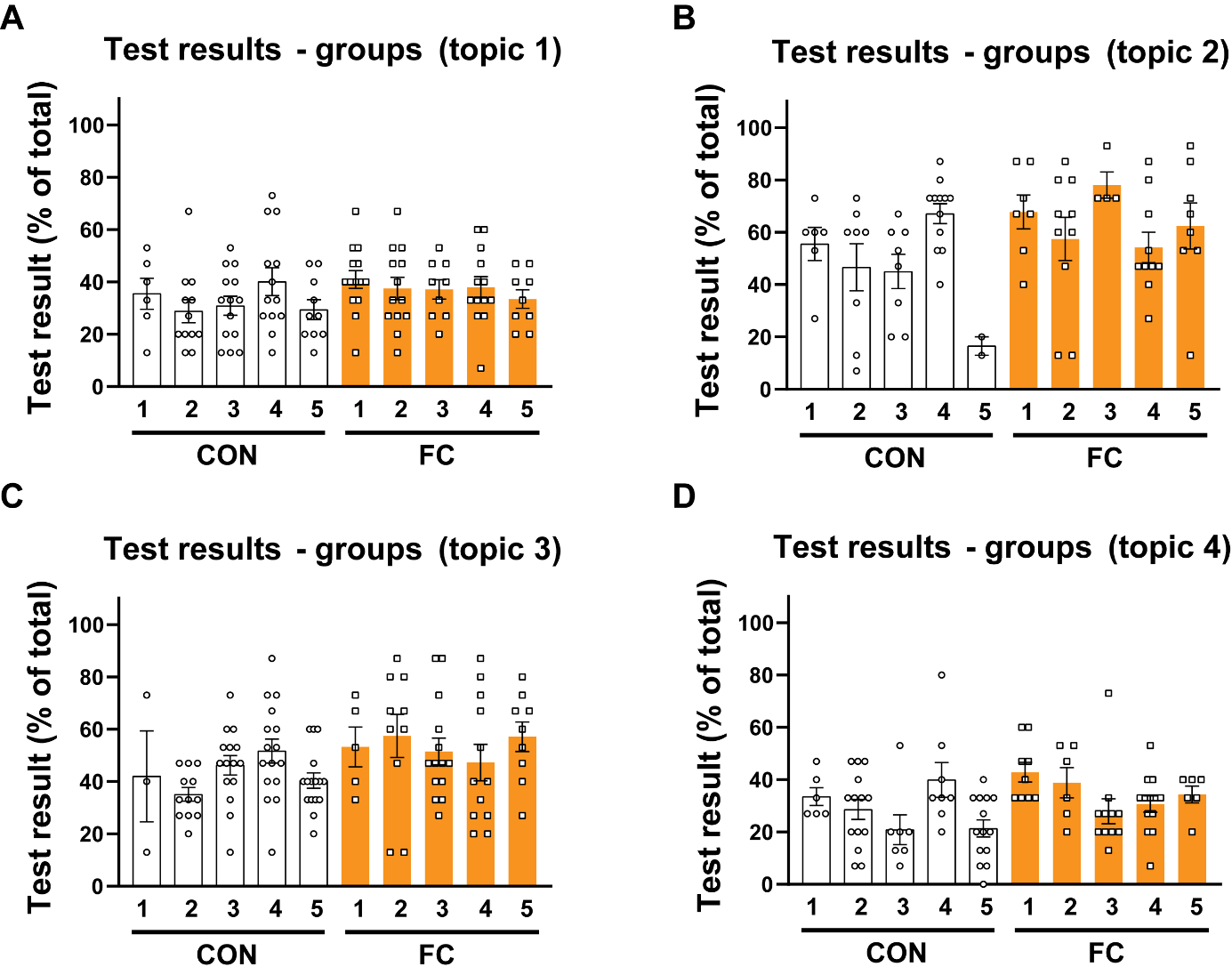
**

**Supplementary Figure 2 – The impact of flipped classroom on student performance in long term. Subgroup analysis was performed with Chi-square test (see Supplementary Table 6)**

**
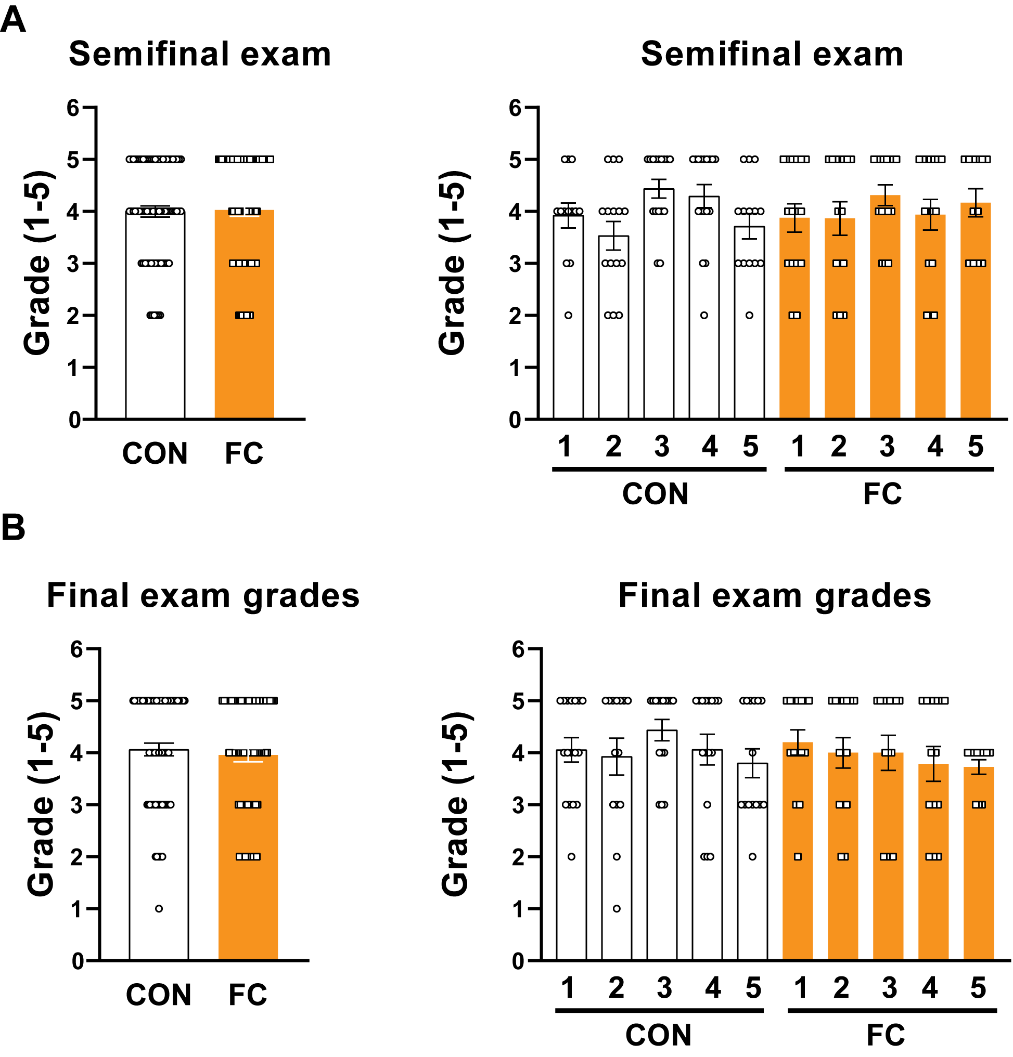
**

**Supplementary Figure 3 – The impact of flipped classroom on student general performance. P>0.05 CON vs FC, Mann-Whiteny test.**
